# RNA denaturation underlies circular RNA separation

**DOI:** 10.1093/nar/gkaf1160

**Published:** 2025-11-20

**Authors:** Yanyi Jiang, Jørgen Kjems

**Affiliations:** Interdisciplinary Nanoscience Center (iNANO), Aarhus University, 8000 Aarhus, Denmark; Interdisciplinary Nanoscience Center (iNANO), Aarhus University, 8000 Aarhus, Denmark; Department of Molecular Biology and Genetics (MBG), Aarhus University, 8000 Aarhus, Denmark

## Abstract

*In vitro*-synthesized circular RNAs (circRNAs) have emerged as a promising drug modality for RNA therapeutics due to their improved stability and reduced immunogenicity. However, effective analysis and purification of circRNAs pose critical challenges arising from the insufficient separation of circRNAs and linear RNA byproducts. In this study, we systematically evaluate the effectiveness of gel electrophoresis and high-performance liquid chromatography–size exclusion chromatography (HPLC–SEC) for separating circRNAs synthesized through ligase- or ribozyme-based strategies. While the synthesis strategy dictates the purification complexity, we demonstrate that both techniques rely on RNA denaturation to successfully separate circRNAs. Additionally, when using HPLC–SEC, we show that even a trace amount of magnesium ions in RNA samples can significantly compromise circRNA separation. Under optimized denaturing conditions, HPLC–SEC enables circRNA purification directly from crude enzymatic reactions, thereby streamlining the purification process. Our study provides mechanistic insights into circRNA separation, advancing the purity and scalability of circRNA-based therapeutics.

## Graphical Abstract

**Graphical Abstract:**
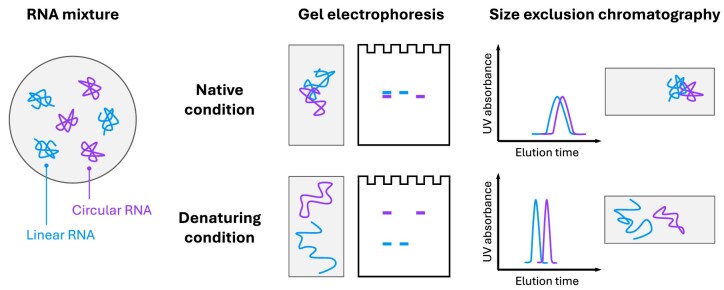

## Introduction

Following the triumph of messenger RNA (mRNA) therapeutics against COVID-19, circular RNAs (circRNAs) have garnered significant interest as a promising modality for nucleic acid therapeutics [1, 2]. circRNAs are single-stranded RNAs with a covalently closed loop structure, lacking free ends. The circular structure renders circRNAs resistant to exonuclease-mediated degradation, providing them with greater stability than their linear counterparts [3]. This enhanced stability makes circRNA an appealing template for potent long-term translation [4–7]. Beyond protein production, the versatile therapeutic potential of circRNAs has been explored in applications such as microRNA sponges, protein sequestration, and gene editing [1]. Another advantage of synthetic circRNAs is their reduced immunological profile. Rigorously purified, unmodified circRNA can evade cellular immune surveillance [6, 8–10].

**Figure 1.**
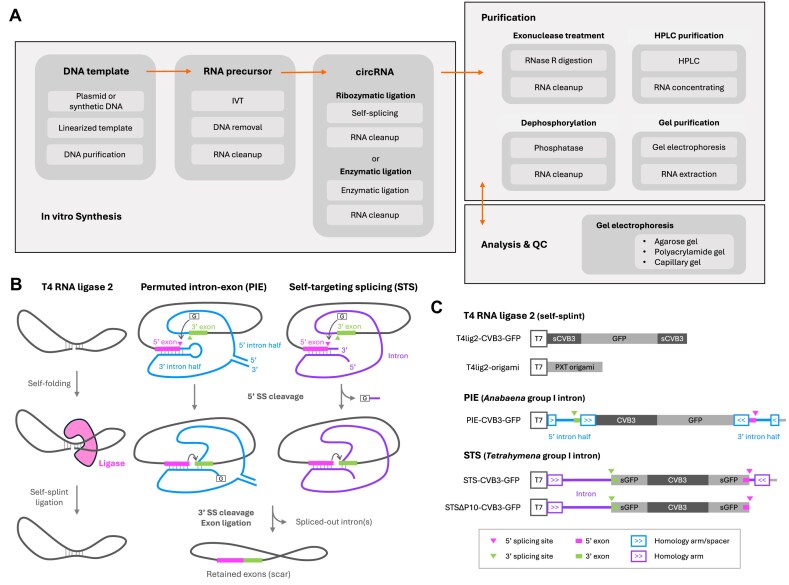
Construct design for circRNA separation investigation. (**A**) Workflow for producing *in-vitro* synthesized circRNAs. (**B**) Mechanism of *in vitro* circRNA synthesis. For enzymatic circularization, T4 RNA ligase 2 recognizes a self-scaffolded double-stranded RNA precursor and seals the nick without requiring an external splint, resulting in the formation of a circRNA. For group I intron-catalyzed circularization, the 5′ and 3′ exons are ligated through self-splicing in either a *cis* or a trans manner, referred to as permuted intron-exon (PIE) or self-targeting splicing (STS), respectively. Both self-splicing strategies undergo two consecutive transesterification reactions, marked with arrows. In the first step, an intron-harbored exogenous guanosine cleaves the 5′ splicing site (SS). In the second step, the 5′ exon and 3′ exon are brought into proximity by conformational change and base pairing to the internal guiding sequence. The 3′-OH group of 5′ exon terminus reacts with the 3′ splicing site, resulting in ligated exons and spliced-out introns. The exon sequences are incorporated into the circRNA, forming a “scar.” G, exogenous guanosine. (**C**) The IVT template design in this study. The CVB3–GFP and PXT origami were the backbones for circRNA synthesis through the three methods described in panel (B). The CVB3 and GFP were split in the T4lig2–CVB3–GFP and STS(∆P10)–CVB3–GFP constructs, designated as sCVB3 and sGFP, respectively. The PIE–CVB3–GFP construct utilized the original *Anabaena* exon sequences for self-splicing, in addition to homology arms and spacers to enhance the reaction, resulting in a scar of 176 nt. The STS constructs embedded exon sequences into sGFP, producing “scarless” circRNA products. The downstream sequence of the 5′ exon in the STS–CVB3–GFP construct was removed to create the STS∆P10–CVB3–GFP construct. T7, T7 promoter.

Producing *in vitro*-synthesized circRNA entails three major steps: synthesis, purification, and analysis with quality control (Fig. 1A). After the synthesis of linear RNA precursors by *in vitro* transcription (IVT), circularization is succeeded through intramolecular ligation using chemical, enzymatic, or ribozymatic approaches (see reviews [1, 11, 12]; Fig. 1B). Although various methods have been developed for circRNA synthesis, large-scale purification remains a significant challenge. Current purification strategies include exonuclease treatment for the selective removal of linear RNA impurities [13], phosphatase treatment to neutralize the immunogenic triphosphate group [6], and gel or HPLC purification to resolve circRNAs based on their physicochemical attributes (Fig. 1A). However, the physicochemical similarity of circular and linear RNA causes them to migrate similarly when analyzed by gel electrophoresis. Given that this method serves as the cornerstone of circRNA analysis methodologies (Fig. 1A), insufficient separation complicates the quality control process, potentially undermining the reliability and reproducibility of circRNA research.

Gel electrophoresis remains the most widely used method for separating circRNAs. Depending on the gel matrix type, it includes agarose gel electrophoresis (AGE), polyacrylamide gel electrophoresis (PAGE), and capillary gel electrophoresis (CGE). In native AGE, linear and circular RNAs tend to overlap [14], while denaturing formaldehyde agarose gels, commonly used for northern blotting, facilitate circRNA separation [15]. Notably, the commercial precast agarose E-Gel system allows permutated intron-exon (PIE) generated circRNAs to be separated from precursors and nicked byproducts [5, 6, 16], though with limited resolution compared to urea PAGE [17, 18]. The best circRNA separation resolution is achieved using denaturing urea PAGE, where circRNAs migrate distinctly slower than their linear counterparts [19]. In contrast, native PAGE provides poor separation efficiency for circRNAs [20, 21]. CGE, commonly applied for rapid RNA quality assessment, is also employed for circRNA quality evaluation [18, 22–27]. While this technique can distinguish PIE-generated circRNAs from their longer precursors, its ability to separate circRNAs from linear RNA counterparts of the same size remains controversial [28]. Among these three electrophoresis methods, both AGE and PAGE can serve for circRNA purification [5, 8], but the inefficiency of the downstream gel extraction of the circRNA limits their application for large-quantity manufacturing.

In contrast, high-performance liquid chromatography–size exclusion chromatography (HPLC–SEC) offers scalability for the purification of biopharmaceutical substances [29] and has been utilized for circRNA purification since 2018 [5]. In HPLC–SEC, circRNAs elute later than their linear counterparts, allowing for their separation [5]. However, the separation remains incomplete as the circRNA fraction partially overlaps with the linear fraction. To obtain relatively pure circRNA, the latter portion of the circRNA fraction is typically collected, although this approach compromises the yield [6, 7, 30, 31]. Factors influencing SEC performance in circRNA purification have not been systematically studied but may include column particle pore size, circRNA size, circRNA synthesis method, and mobile phase conditions (reviewed in Table 1). Of note, the choice of analysis and quality control methodology directly impacts the purity assessment of circRNAs.

**Table 1.** Literature summary of circRNA HPLC–SEC purification

Year	Synthesis method(s)	circRNA length (~nt)	SEC column (brand, pore size, particle size)	Mobile phase	circRNA analysis	Reference
2018	PIE-Ana	1500	Sepax, 2000 Å, 5 µm	TE, pH 6.0	AGE (E-Gel)	[5]
2019	PIE-Ana	1500	Sepax, 2000 Å, 5 µm	TE, pH 6.0	AGE (E-Gel)	[6]
2019	PIE-td	1500	Sepax, 2000 Å, 5 µm	TE	AGE, CGE	[32]
2020	PIE-Ana	350 to 500	Sepax, 2000 Å, 5 µm	TE, pH 6.8	AGE (E-Gel)	[30]
2022	PIE-Ana, T4lig2	1600 to 1900	Sepax, 2000 Å, 5 µm	TE, pH 7.5	AGE, urea PAGE	[7]
2022	T4lig1, T4lig2	150	Sepax, 2000 Å, 5 µm	TE	AGE	[33]
2022	PIE-Ana	1100 to 1400	Sepax, 2000 Å, 5 µm	TE, pH 6.0, 35°C	AGE (E-Gel)	[16]
2022	PIE-Ana, PIE-td, T4lig1	300 to 550	Sepax, 2000 Å, 5 µm	TE, pH 6.0	AGE, urea PAGE	[34]
2022	PIE-Cte	1500	Waters XBridge, 450 Å, 3.5 µm; Sepax, 1000 Å, 5 µm	TE, PB, pH 7.4, 25°C	AGE	[35]
2022	PIE-Ana	1800	Sepax, 1000 Å, 5 µm	PB, pH 6.0	AGE, CGE	[36]
2022	PIE-Ana	1700 to 2700	Sepax, 2000 Å, 5 µm	TE, pH 6.0	AGE, CGE	[31]
2024	PIE-Ana	1500	Sepax, 2000 Å, 5 µm	not specified	AGE	[37]
2024	PIE-Ana	2700	Sepax, 2000 Å, 5 µm	TE, pH 6.0	AGE	[38]
2024	PIE-Ana	1600	Sepax, 1000 Å, 5 µm	PB, pH 7.0	CGE, RP-HPLC	[28]
2024	PIE-Ana, PIE-td	1500	Sepax, 1000 Å, 5 µm	PB, pH 7.0	AGE	[39]
2024	TRIC-Ana	1400	Sepax, 2000 Å, 5 µm	TE, pH 6.5	(urea) AGE	[10]
2025	PIE-Ana	1600	Sepax, 1000 Å, 5 µm	PB, pH 6.0	CGE	[40]
2025	PIE-Ana	520	Agilent, 2000 Å, 5 µm	TE, pH 6.0	AGE (E-gel)	[41]
2025	Chemical	760	Yarra, 4000 Å, 3 µm	PB, pH 6.8, 25°C	urea PAGE	[42]

T4lig1: T4 RNA ligase 1. T4lig2: T4 RNA ligase 2. PIE: permuted intron-exon. TRIC: *trans*-ribozyme-based circularization. Ana: *Anabaena* pre-tRNA group I intron. td: bacteriophage T4 thymidylate synthase gene group I intron. Cte: *Clostridium tetani* group II intron. TE: Tris–EDTA buffer, 10 mM Tris–HCl, 1 mM EDTA. PB: Phosphate buffer with variable ratio of NaH_2_PO_4_ and Na_2_HPO_4_. AGE: agarose gel electrophoresis. PAGE: polyacrylamide gel electrophoresis. CGE: capillary gel electrophoresis. RP-HPLC: reverse-phase HPLC.

In this study, we have conducted a comprehensive investigation to evaluate the separation effectiveness of circRNA using gel electrophoresis and HPLC–SEC, revealing that RNA denaturation is critical for effective circRNA separation. Comparing circRNAs synthesized through enzymatic and ribozymatic methods, we highlight the purification complexities arising from the differing byproduct profiles and sample processing associated with each synthesis method.

## Materials and methods

### *In vitro* transcription

The sequences of PIE-, STS-, and self-splint T4lig2–CVB3–GFP were obtained from previous published studies [5, 7, 43]. The DNA templates were synthesized by Integrated DNA Technologies or Twist Bioscience (sequences shown in Supplementary Table S1). The IVT templates were linearized and amplified through polymerase chain reaction using Q5 DNA polymerase (NEB), followed by agarose gel purification using Monarch DNA Gel Extraction Kit (NEB). RNAs were *in vitro* transcribed using the HiScribe T7 Quick High Yield RNA Synthesis Kit (NEB). Specifically, 100–500 ng purified DNA template was added into 20 μl reaction volume and incubated at 37°C overnight. To produce the RNA precursor with 5′ monophosphate for T4 ligation, additional GMP (Sigma) was added (50 mM final concentration) to the IVT reaction. After incubation, the IVT sample was diluted to 50 µl using RNase-free water and treated with 2 µl DNase I (NEB) at 37°C for 15 min. The RNA was subsequently column-purified using the MEGAclear Transcription Clean-Up Kit (Invitrogen) according to the manufacturer’s instructions.

### RNA circularization

For T4 ligation constructs, column-purified linear RNA precursors were incubated with T4 RNA ligase 2 (NEB) following the manufacturer’s protocol at 37°C for 2 h. For the PIE construct, 5 µl 10× T4 RNA ligase buffer (500 mM Tris–HCl, 100 mM MgCl_2_, 10 mM DTT, pH 7.5; NEB) and GTP (final concentration of 2 mM) were added to column-purified RNA samples, bringing the total volume to 50 µl, followed by incubation at 55°C for 15 to 30 min. After the circularization process, RNAs were column-purified by RNA Clean & Concentrator-25 (Zymo Research) for subsequent studies. For STS constructs, no circularization step was performed after DNase I treatment [17].

### Enzymatic treatments for circRNA analysis

RNase R (Biosearch Technologies) treatment was performed after RNA cleanup at 1 U/µg RNA, with incubation at 37°C for 15 to 60 min. In the control groups, the RNase R was replaced by RNase-free water. Since the performance of RNase R can vary between batches and RNA constructs, conducting a pilot test and optimizing incubation time and enzyme concentration are necessary. Following RNase R treatment, RNA was directly subjected to electrophoresis analysis without column purification.

For the RNase H cleavage assay, 0.5–3 µg of column-purified RNAs were incubated at 37°C for 15 min with DNA probes in RNase H buffer (Qiagen). The RNA:DNA molar ratio was 1:2 (probe sequences are listed in Supplementary Table S2). After the initial incubation, RNase H (Qiagen) was added at 1 U per µg of RNA, and reactions were incubated at 37°C for an additional 30 min. Reaction products were analyzed by gel electrophoresis without RNA cleanup.

### RNA crosslinking

Prior to crosslinking, linear RNA was purified by HPLC–SEC, and circRNA was enriched by RNase R treatment. Crosslinking was performed following a published protocol [44] with minor modifications. Briefly, 900 ng of purified RNA was mixed with 1.2 µl 4′-amino-methyl trioxsalen hydrochloride (AMT; 1 mg/ml in water; Santa Cruz) in 3 µl 10× RNase R buffer [0.2 M Tris–HCl (pH 8.0), 1 M KCl, 1 mM MgCl_2_; Biosearch Technologies] in a total volume of 30 µl, yielding a final AMT concentration of 40 µg/ml. RNA samples were kept on ice during preparation and protected from light. The mixtures were then irradiated on ice with 365 nm light in a UV oven (CL-1000 Ultraviolet Crosslinker, UVP). Samples were positioned 2–3 cm from the UV lamp without any barrier. Aliquots were collected after 0, 5, 15, 30, and 60 min of UV exposure, mixed with 2× RNA loading dye (NEB), and analyzed by gel electrophoresis. For HPLC analysis, RNA samples were irradiated for 60 min and subsequently purified using the RNA Clean & Concentrator-5 kit (Zymo Research) before HPLC injection.

### Agarose and polyacrylamide gel electrophoresis

Self-cast agarose gels were prepared by dissolving 1% or 2% (w/v) agarose (UltraPure, Invitrogen) in 1× TBE buffer [100 mM Tris, 90 mM boric acid, and 1 mM ethylenediaminetetraacetic acid (EDTA), pH 8.3], diluted from UltraPure TBE 10× stock (Invitrogen) with ultrapure water. Before gel solidification, 3–5 µl SYBR Safe (Invitrogen) was pre-mixed into 100 µl of the melted gel. RNA samples were mixed with a denaturing 2× RNA Loading Dye (95% formamide, 0.02% SDS, 0.02% bromophenol blue, 0.01% xylene cyanol, 1 mM EDTA; NEB), followed by snap cooling (90°C for 1 min, then on ice for at least 5 min). The gel was run in 1× TBE buffer using 150 to 200 V at room temperature.

For the E-Gel electrophoresis system, three types of E-Gels (Invitrogen) were used: 1% non-EX gel (A45203), 2% non-EX gel (A45205), and 2% EX gel (G402022). RNA samples (50–1000 ng) were diluted to 20 μl with nuclease-free water and loaded onto precast gels without prior snap-cooling. Electrophoresis was conducted using E-Gel Power Snap Electrophoresis Device (Invitrogen), following either the “E-Gel 1%–2% EX” program for 20 min or the “E-Gel 0.8%–2%” program for 20 to 40 min. The temperatures of E-Gels were measured immediately after electrophoresis using a miniature infrared thermometer (Elma 608, Elma Instruments).

For denaturing PAGE, urea polyacrylamide gels were prepared using the UreaGel System Kit (National Diagnostics), where UreaGel concentrate (19:1 acrylamide/bisacrylamide, 7.5 M urea) was mixed with UreaGel diluent (7.5 M urea) at varying ratios depending on the desired gel percentage and TBE buffer (1× final concentration, pH 8.3). For polymerization, 80 µl 10% (w/v) APS and 4 µl TEMED were added to a 10 ml gel solution. RNA samples or HPLC fractions were mixed with the denaturing 2× RNA Loading Dye (NEB), followed by snap cooling (90°C for 2 min, then placed on ice for at least 5 min). Electrophoresis was performed in 1× TBE buffer at 350 V and 20 W for 20–30 min at room temperature.

For native PAGE, AccuGel premixed solution (40% (w/v), 29:1 acrylamide/bisacrylamide, National Diagnostics) was diluted with ultrapure water and TBE buffer (1× final concentration, pH 8.3) to prepare the gel. For polymerization, 80 µl 10% (w/v) APS and 4 µl TEMED were added to a 10 ml gel solution. RNA samples were combined with a non-denaturing 6× loading dye (TrackIt Cyan/Yellow Loading Buffer, 10 mM Tris–HCl, 60 mM EDTA, 0.03% xylene cyanol FF, 0.3% tartrazine, 60% glycerol, pH 7.6; Invitrogen) before gel loading. Electrophoresis was carried out in a 4°C cold room using 1× TBE as the running buffer at 80 V for 130 min.

All polyacrylamide gels were prepared using mini gel cassettes (8 × 8 cm, 1.0 mm, Invitrogen), and the electrophoresis was performed using XCell SureLock Mini-Cell (Invitrogen). After electrophoresis, the polyacrylamide gels were stained with SYBR Gold (Invitrogen) to visualize RNA bands. The ssRNA ladder (NEB) was used as the size marker. Both agarose and polyacrylamide gels were visualized by an Amersham Typhoon biomolecular imager (Cytiva).

### Capillary gel electrophoresis

RNA samples (1 µl) were loaded onto a microfluidic chip from the RNA 6000 Nano Kit (Agilent, 5067-1511) following the manufacturer’s instructions. For the pre-denaturing condition, before loading, RNA samples were mixed with the denaturing 2× RNA Loading Dye (NEB), heated at 65°C for 2 min, and snap-cooled on ice. The samples were analyzed using the 2100 Bioanalyzer (Agilent).

### HPLC–SEC

RNA samples (1–5 µg) were injected into a size exclusion column on an Agilent 1100 Series HPLC (Agilent), with RNase-free TE buffer (10 mM Tris, 1 mM EDTA, pH 7.5; 20× stock, Thermo Fisher) as mobile phase at a flow rate of 0.3 ml/min (SEC-1000/-2000) or 0.7 ml/min (SEC-4000). The SEC columns are with particle size of 5 μm and pore size of 2000 Å (4.6 × 300 mm; Sepax, 215980P-4630) or 1000 Å (4.6 × 300 mm; Sepax, 215950P-4630) or 4000 Å (8 × 300 mm; Altmann Analytik, AANRS4SE-5030080). The SEC-2000 and SEC-1000 columns were wrapped with copper wires and connected to an extended stainless steel capillary (0.17 × 450 mm, Agilent) in the inlet. The capillary was attached to a heating plate to preheat the mobile phase before it entered the column (Supplementary Fig. S2A) [45]. RNA was detected by UV absorbance at 260 nm. Collected HPLC fractions were analyzed by 2% E-Gels or 3.5% urea PAGE, or concentrated using the RNA Clean & Concentrator-25 kit (Zymo Research) for subsequent RNA crosslinking. The separation resolution (*R*_s_) was calculated using the following equation:

${{R}_s} = 1.176\ast ({{{{t}_{R2}} - {{t}_{R1}}}})/({{{{W}_{1/2}}1 + {{W}_{1/2}}2}}),$ where *t_R1_* and *t_R2_* are the retention times of the two peaks, and *W*_1/2_1 and *W*_1/2_2 represent the peak widths at half height for each peak.

## Results

### Analysis of circRNAs with gel electrophoresis

To investigate how circRNA synthesis methods affect the subsequent analysis in gel electrophoresis, we designed five constructs where two different circRNAs are produced using three different circularization methods (Fig. 1C). Three of the constructs contain the commonly utilized coxsackievirus B3 (CVB3) internal ribosome entry site (IRES) upstream of GFP open reading frame: (i) T4 RNA ligase 2 mediated self-splint ligation (T4lig2–CVB3–GFP) [7, 46]; (ii) permuted intron-exon (PIE) *Anabaena* group I intron mediated circularized through *cis* self-splicing (PIE–CVB3–GFP) [5, 47]; and (iii) self-targeting splicing (STS) *Tetrahymena* group I intron mediated circularization (STS–CVB3–GFP) [17, 43]. A variant of the latter construct was also included (STSΔ10–CVB3–GFP) where the downstream sequence of the 5′ splicing site was deleted. This version exhibits higher RNA circularization efficiency, likely due to the omission of the first esterification reaction leading to 5′ splicing site cleavage (Fig. 1B) [17]. As an example of a shorter (415 nt) circRNA without translation elements, we used the paranemic crossover triangle (PXT) RNA origami [48] adopted for T4 RNA ligase 2 mediated self-splint ligation (T4lig2-origami).

The product profiles differ depending on circRNA synthesis strategies. T4 enzymatic ligation primarily produces unreacted precursors of the same size as circRNAs, while ribozyme-catalyzed circularization involves longer precursors, potential splicing intermediates, and excised introns (Fig. 1B). Common to all strategies, inevitable circRNA nicking leads to linear side products, either matching the size of circRNAs or shorter if multiple nicks occur.

**Figure 2.**
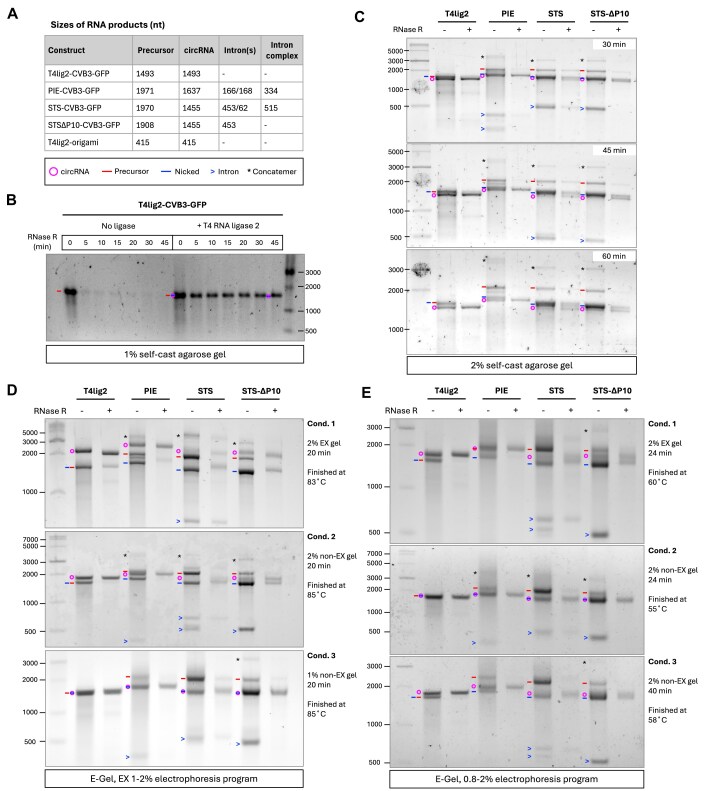
Agarose gel analysis of circRNAs. (**A**) The predicted sizes of RNA products formed upon the circularization of the different constructs. Symbols indicating the gel bands are circRNA (magenta circle), precursor (red line), nicked circRNA (blue line), spliced intron (blue arrow), and concatemer (asterisk). The RNA species are denoted according to RNA sizes and RNase R sensitivity. (**B**) Analysis of products in a 1% self-casted agarose gel. The RNA precursor from T4lig2–CVB3–GFP was incubated with or without T4 RNA ligase 2, followed by RNase R treatment (0 to 45 min) and subsequent gel electrophoresis. (**C**) Analysis of products in a 2% self-cast agarose gel. The four denoted RNA constructs were treated with RNase R and analyzed using the indicated electrophoresis running times. (**D**) E-Gel analysis of circRNAs in 1% or 2% E-Gels using the “E-Gel EX 1–2%” program. The four denoted RNA constructs were treated with or without RNase R before loading. The surface temperatures of E-Gels were measured immediately after the electrophoresis was done. (**E**) E-Gel analysis of the same samples as in panel (D) but using the “E-Gel 0.8%–2%” program.

Native agarose gel is commonly employed to analyze synthetic circRNAs. Here, we investigated the ability of 1% and 2% self-cast and commercial precast agarose E-Gels to separate circRNA from linear contaminates (Fig. 2). For all reactions, the exonuclease RNase R was used to identify the bands corresponding to circRNAs. Self-cast 1% agarose gels were unable to separate circular and linear CVB–GFP RNAs of the same size (Fig. 2B). In self-cast 2% agarose gel, circRNA migrates slightly faster than its linear counterpart, but the resolution is very limited, as the linear and circRNA bands remain close, even after extended electrophoresis (Fig. 2C). The commercial precast agarose E-Gel has been reported to separate circRNA from its linear counterpart [5, 6, 16]. There are two types of E-Gels, EX and non-EX, which can be run using various electrophoresis programs. Using an EX 1%–2% electrophoresis program for 20 min, both 2% EX and 2% non-EX gels could separate circRNAs from linear ones, but 1% non-EX gel could not (Fig. 2D). Using a non-EX 0.8%–2% electrophoresis program, circRNA could be separated by two types of 2% E-Gels but with lower resolution, and non-EX gel required extended electrophoresis to achieve circRNA separation (Fig. 2E). Notably, after electrophoresis, the gel temperature differed among programs and E-Gel types. EX and non-EX gels reached above 80°C with EX 1%–2% program after 20-min electrophoresis, whereas the gel temperature only reached ~55°C with non-EX 0.8%–2% program for 24 to 40 min, indicating higher temperature may facilitate circRNA separation.

**Figure 3.**
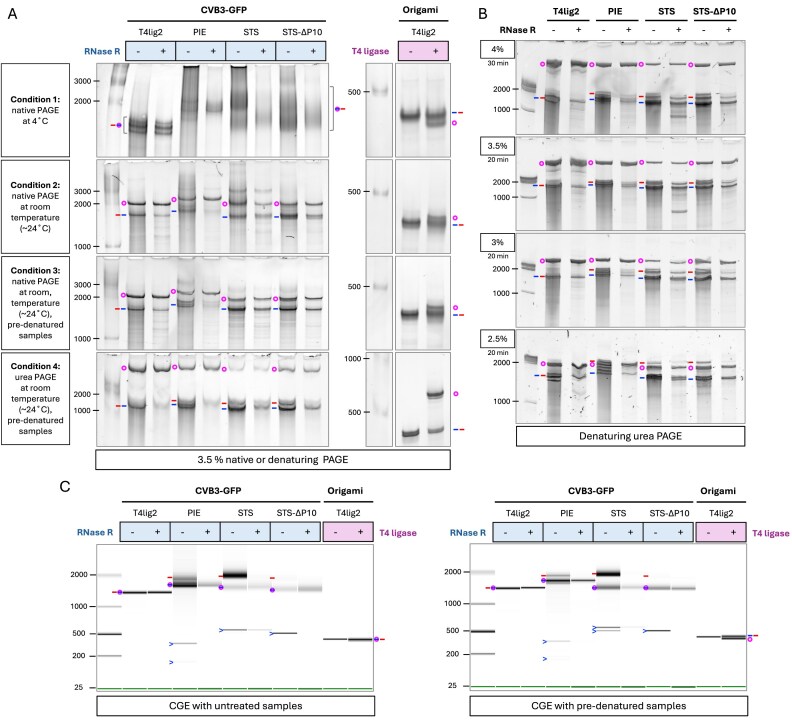
PAGE and CGE analysis of circRNAs. (**A**) PAGE analysis of circRNAs under native and denaturing conditions. The CVB3–GFP circRNA samples (left panel) were incubated with or without RNase R. The origami RNA precursor (right panel) was incubated with or without T4 RNA ligase 2. Four different PAGE conditions were tested: (i) Native PAGE. RNA samples were mixed with native loading dye and analyzed in a 3.5% native polyacrylamide gel at 4°C (80 V for 130 min). (ii) Same as Condition 1, but run at room temperature (350 V for 16 min). (iii) Same as Condition 2 but with RNA samples pre-denatured by mixing with denaturing 2× RNA Loading Dye (NEB), heating at 90°C for 2 min, and then snap-cooling on ice before being analyzed. (iv) Same as Condition 3 but using denaturing PAGE (7.5 M urea). (**B**) Denaturing PAGE analysis of circRNAs in gels at indicated acrylamide concentrations. Pre-denatured RNA samples were analyzed by 7.5 M urea PAGE at 350 V for 20 or 30 min at room temperature. (**C**) CGE of circRNAs. Products from the denoted RNA constructs were analyzed using the 2100 Bioanalyzer (Agilent). For the pre-denaturing condition (right), RNAs were mixed with 2× denaturing RNA loading dye, heated at 65°C for 2 min, and then snap-cooled on ice prior to chip loading. The gel bands are annotated with symbols as defined in Fig. 2A.

Denaturing urea PAGE provides superior circRNA separation efficacy, where circRNAs migrate markedly slower than linear ones [19]. In contrast, native PAGE has difficulties separating linear and circular RNAs, with circRNAs either migrating similarly or slightly faster than their linear forms [20, 21]. First, we investigated how gel conditions and sample pretreatment affect the separation of CVB3–GFP and PXT origami-derived circRNAs (Fig. 3A). Given that the linear RNA precursor of T4lig2-origami resisted RNase R digestion because of its compact 3′ end structure [49], we chose the T4 ligase-untreated group as the linear control. For native 3.5% PAGE performed at 4°C, circular T4lig2-origami (415 nt) migrated slightly faster than its linear form (Fig. 3A, Condition 1), which aligns with the former finding [21]. Under native conditions, the circRNA separation of CVB3–GFP constructs (1450–1650 nt) was poor. All three ribozyme-derived groupsde showed smeared circRNA bands (Fig. 3A, Condition 1), suggesting the presence of multiple RNA conformations. In contrast, T4lig2-origami appeared as a sharper band in the native gel analysis, probably due to its smaller size and lower structural complexity. When the PAGE was processed at room temperature, circRNAs moved slower than linear counterparts in the gel (Fig. 3A, Condition 2). Pre-denatured samples with 47.5% formamide and snap cooling before gel loading further improved separation and resolution of ribozyme-synthesized circRNAs, yielding sharper circRNA bands and reduced smearing (Fig. 3A, Condition 3). In 7.5 M urea denaturing PAGE, circRNAs migrated even slower, and smearing bands appearing in native gels were sharpened, resulting in greater separation efficiency (Fig. 3A, Condition 4). These results demonstrate how RNA denaturation, in general, promotes circRNA separation from linear counterparts. Next, we tested circRNA migration under varying gel concentrations in denaturing PAGE (Fig. 3B). Reducing the gel concentration from 4% to 2.5% lowered the separation efficiency for circRNAs. In 2.5% denaturing PAGE, STS(∆P10)–CVB3–GFP circRNAs even migrated faster than the linear precursors. Considering both electrophoresis time and separation efficiency, we selected 3.5% urea PAGE for the subsequent analysis of PIE and STS constructs.

In addition to exonuclease treatment, we validated RNA circularization through RNase H assays with DNA probes complementary to either the junction or non-junction region (Supplementary Fig. S1A). With junction-targeting probes, RNase H specifically degraded STS∆10–CVB3–GFP circRNA, whereas T4lig2–CVB3–GFP and PIE–CVB3–GFP circRNAs were resistant (Supplementary Fig. S1C), likely because their junctions, located within the IRES or spliced exon regions, are structurally inaccessible to DNA probes or RNase H (Supplementary Fig. S1B). To address this, RNase H digestion was performed following RNase R treatment. In the presence of a non-junction-targeting probe, RNase H cleaves circRNAs into linear forms, producing a major band corresponding in size to the expected nicked product, consistent with the annotation of the circular species. Specificity and activity of RNase H were confirmed by cleaving linear precursors (generated in the absence of ligase or by ribozyme mutation), which yielded two smaller fragments of the predicted sizes (Supplementary Fig. S1D).

Next, we investigated the circRNA separation using CGE with and without pre-denaturing the samples before loading (Fig. 3C). Under native conditions, CGE failed to separate the T4lig2–CVB3–GFP and T4lig2-origami circRNAs when the circRNAs share the same size as their linear precursors (Fig. 3C, left panel). However, when samples were pre-denatured with formamide and snap-cooled, the circRNA from T4lig2-origami exhibited slightly faster migration than the linear precursor (Fig. 3C, right panel). Additionally, we observed that pre-denaturation resulted in sharper bands in CGE, possibly attributed to increased conformational homogeneity of RNA following the denaturing process. Since the circRNA yield was quite low from the STS–CVB3–GFP construct (Fig. 3B), we opted for STS∆P10–CVB3–GFP in our subsequent studies.

### Column heating enables effective separation of circRNAs in HPLC–SEC

Inspired by the observation that RNA denaturation during electrophoresis is crucial for effective circRNA separation, we hypothesized that this principle can be utilized in HPLC–SEC. When heating the SEC column, we noticed that the air temperature of the HPLC column oven was lower than the setting (Supplementary Fig. S2A). To ensure the SEC column constantly stayed at the pre-set temperature, we wrapped the column with a copper wire to facilitate better heat conduction and attached extended tubing to the heating plate to preheat the mobile phase before it entered the column [45] (Supplementary Fig. S2A).

**Figure 4.**
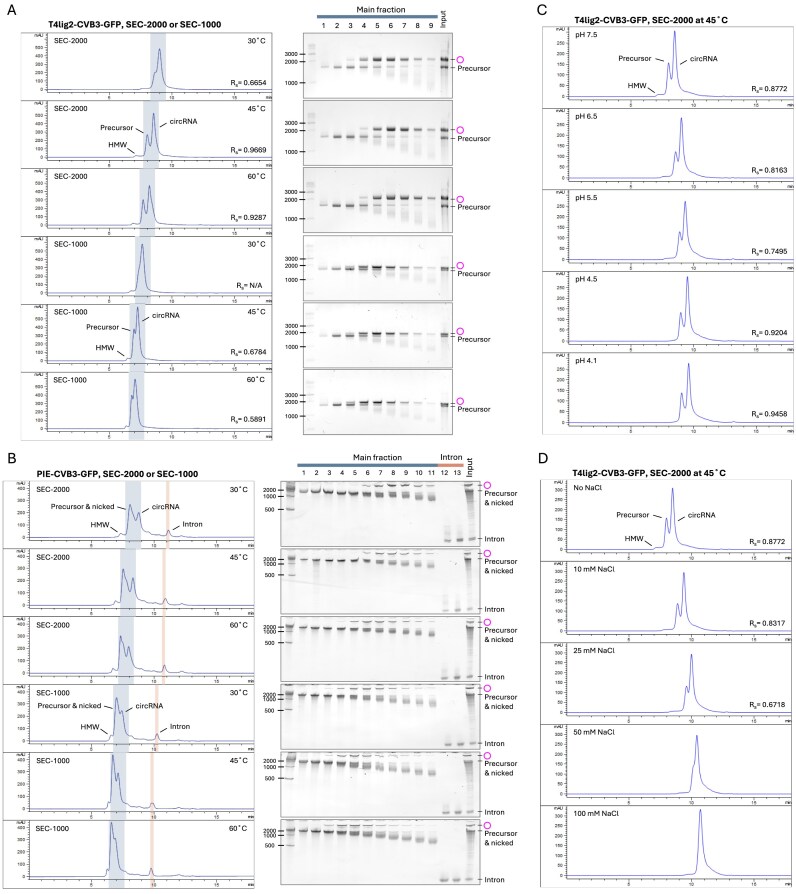
HPLC–SEC purification of circRNAs. (**A**) Separation of T4lig2–CVB3–GFP-derived products in SEC-1000/-2000 at different column temperatures. RNAs were purified with SEC-1000 or -2000 at 30°C, 45°C, and 60°C. The eluted fractions (marked in blue) were analyzed using E-Gels (2% EX gel for SEC-2000 elutes, 2% non-EX gel for SEC-1000 elutes; EX 1%–2% program, 20 min). HMW, high molecular weight. (**B**) Same as in panel (A), but analyzing the separation of PIE–CVB3–GFP–derived products by 3.5% urea–PAGE at the indicated temperature. Intron fractions are depicted in red. (**C**) T4lig2–CVB3–GFP-derived RNA separated by the SEC-2000 column at 45°C, with mobile phases adjusted to the indicated pH. (**D**) T4lig2–CVB3–GFP-derived RNA was purified using the SEC-2000 column at 45°C with mobile phases containing the indicated salt concentrations.

We first tested circRNA separation in SEC-2000 (2000 Å pore size, 5 µm particle size) using the T4lig2–CVB3–GFP construct and analyzed the elution fractions by E-Gel electrophoresis (Fig. 4A). The separation resolution (*R*_s_) was measured by both retention time and peak width at half height (see the “Materials and methods” section), where higher *R*_s_ value indicates better separation between two peaks. We found that the circRNA peak largely overlapped with the precursor peak at a column temperature of 30°C. However, heating the column to 45°C or 60°C significantly improved the resolution. Column heating also decreased the retention times for both linear and circular RNA fractions, with a more pronounced effect on linear RNAs, resulting in overall better resolution. Next, we tested SEC-1000 (1000 Å pore size, 5 µm particle size) using the same samples and analyzed the eluted fractions by E-Gel electrophoresis. Heating of the SEC-1000 column also resulted in a better circRNA separation, but the resolution was poorer than using the SEC-2000 column (Fig. 4A).

As expected, the HPLC chromatographic profiles of PIE and STS constructs appeared more complicated than those of the T4 ligation system. The separation of PIE–CVB3–GFP circRNAs was also enhanced when the column was heated to 45°C, according to the analysis of the fractions by urea PAGE (Fig. 4B). For the STS∆P10–CVB3–GFP construct, we observed a plethora of nicked or non-circularized RNAs. Heating the column from 30°C to 45°C did not significantly increase the resolution, and there was a large overlap of the circRNA and linear peaks at 60°C (Supplementary Fig. S2B). Interestingly, for the STS∆P10–CVB3–GFP construct, spliced-out introns co-eluted with precursor and circRNA fractions at 30°C and 45°C, but were separated at 60°C. Again, SEC-2000 outperformed SEC-1000 in separating the CVB3–GFP circRNA in both PIE and STS systems, consistent with previous observations from the T4 ligation system. We hypothesized larger pore size might facilitate circRNA separation and tested SEC-4000 (4000 Å pore size, 5 µm particle size). However, SEC-4000 failed to differentiate circRNA from precursors and small introns at both 30°C and 65°C (Supplementary Fig. S3).

To assess the impact of RNA size on circRNA separation by HPLC–SEC, we tested the smaller and more structured construct, T4lig2-origami (415 nt). Elevated temperature again improved separation. Compared with the larger CVB3–GFP constructs (~1500 nt, Fig. 4B), the T4lig2-origami circRNA was better separated by SEC-1000, which achieved higher Rs values than SEC-2000 (Supplementary Fig. S4). These findings highlight the importance of selecting appropriate column type and temperature based on RNA size and structure.

### pH and salt in the SEC mobile phase influence circRNA separation

A comprehensive study of circRNA separation in different SEC mobile phases is currently lacking. Therefore, we evaluated the resolution of circRNA separation using a Tris-EDTA buffer with a pH range from 7.5 to 4.1, utilizing SEC-2000 column heated to 45°C. For the T4lig2–CVB3–GFP construct, the separation resolution (*R*_s_) declined when lowering the pH from 7.5 to 5.5, but improved when lowering the pH further from 5.5 to 4.1 (Fig. 4C). A similar trend was seen for the PIE–CVB3–GFP construct (Supplementary Fig. S5A), whereas the difference was not discernible for the STS∆P10–CVB3–GFP construct (Supplementary Fig. S5B).

We then tested how salt concentrations in the SEC mobile phase affect circRNA separation. For the T4lig–CVB3–GFP RNA injected into a SEC-2000 column at 45°C, increasing the sodium chloride concentrations from 0 to 100 mM had a negative effect on circRNA separation efficiency (Fig. 4D). In contrast, for the PIE and STS constructs, low salt conditions (10 mM NaCl) in the mobile phase slightly improved circRNA separation (Supplementary Fig. S6). However, at higher salt concentrations (>25 mM NaCl), the SEC could not separate the circRNAs for any of the constructs.

### Pretreatment of RNA affects circRNA separation in HPLC–SEC

**Figure 5.**
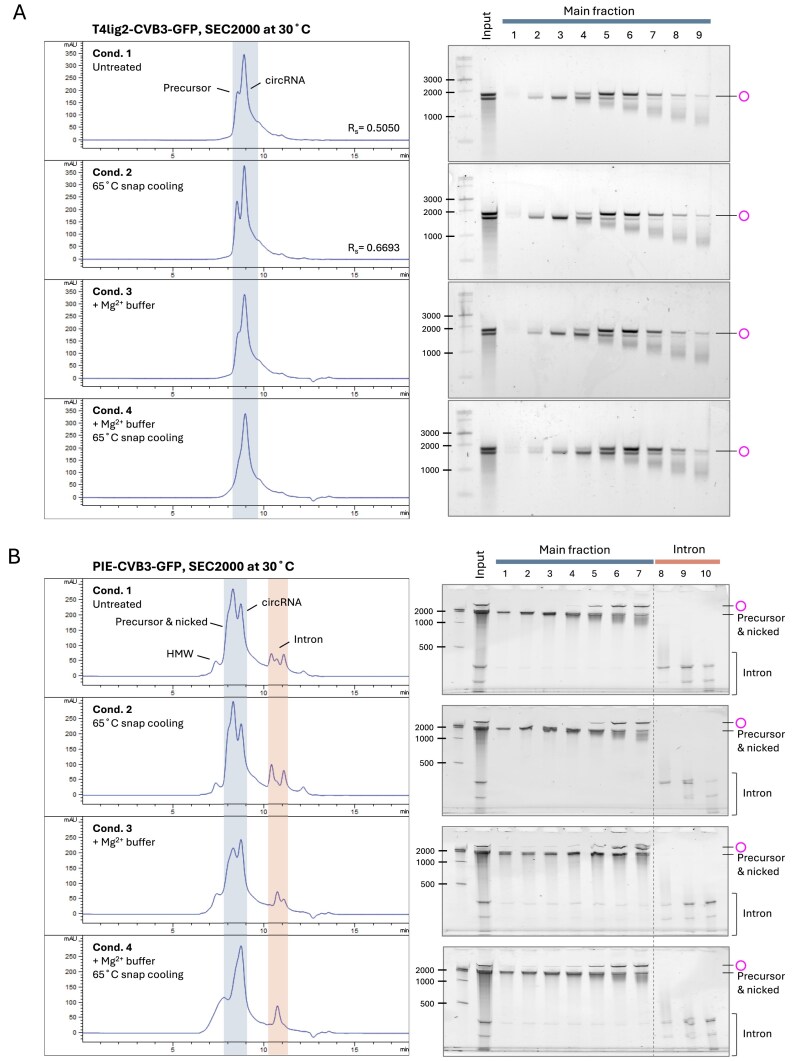
Effect of RNA pretreatment on circRNA separation in HPLC–SEC. (**A**) Effect of the indicated pretreatment of the T4lig1–CVB3–GFP RNAs prior to HPLC–SEC purification. Processed samples were subsequently injected into the SEC-2000 column at 30°C. Condition 1: T4lig2–CVB3–GFP RNA was column-purified after T4 ligation. Condition 2: the cleaned-up RNA was heated to 65°C for 3 min and immediately placed on ice until injection. Condition 3: T4 RNA ligase buffer (NEB) was added to the cleaned-up RNA sample, containing 50 mM Tris–HCl, 10 mM MgCl₂, and 1 mM DTT, pH 7.5. Condition 4: the column-purified RNA was mixed with T4 RNA ligase buffer followed by snap cooling. The main and spliced-out intron fractions, marked in blue and red, respectively, were analyzed by the 2% non-EX gel electrophoresis (EX 1%–2% program, 20 min). (**B**) Conditions as in panel (A) but using column-purified PIE–CVB3–GFP RNA samples and analyzed by 3.5% urea PAGE. Two adjacent intron HPLC fractions were combined and loaded into a single lane for PAGE analysis (lanes 8–10), while the main HPLC fraction was analyzed as single fractions per lane (lanes 1–7).

An RNA cleanup step is typically required prior to HPLC injection to remove unwanted small molecules, such as salts and unincorporated nucleotides, as well as proteins derived from enzymatic reactions (Fig. 1A). We hypothesized that subtle differences in the stringency of the RNA cleanup prior to HPLC injection could affect the chromatographic profiles. Therefore, we investigated how sample treatment and residual materials affect SEC performance for circRNA separation (Fig. 5, Supplementary Fig. S7). We first tested snap cooling of RNA at 65°C before injecting it into the HPLC system. This resulted in a slight increase in the resolution for the T4lig2–CVB3–GFP construct, as both circRNA and precursor peaks became sharper (Fig. 5A, Condition 2). Interestingly, the snap cooling process diminished the co-elution of introns with larger RNA species for both PIE and STS constructs (Fig. 5B, Supplementary Fig. S7, Condition 2). Sufficient magnesium ion concentration is critical for IVT and ribozyme activity. Thus, we examined how adding MgCl_2_ to spin column-purified RNA samples affects SEC performance. The separation of circRNAs, derived from T4 ligation, PIE, and STS constructs, was compromised by the presence of 10 mM Mg^2+^ in RNA samples (Fig. 5A and B, Supplementary Fig. S7, Condition 3). Additionally, more introns co-fractionated with precursors and circRNAs, and more circRNAs were detected in precursor fractions. When snap cooling was applied to Mg^2+^-containing RNA samples, the subsequent HPLC–SEC could not separate circRNAs (Fig. 5A and B, Supplementary Fig. S7, Condition 4). In contrast, the addition of 10 mM EDTA, a strong chelating agent that deactivates divalent metal ions, followed by snap cooling mitigated the issue of intron co-elution in STS∆P10–CVB3–GFP circRNA fractions, resulting in improved circRNA purification (Supplementary Fig. S7, Condition 5).

Elution conditions differ among commercial RNA cleanup kits in terms of temperature and buffer composition. For example, Zymo spin columns use RNase-free water at room temperature, whereas MEGAclear columns require elution buffer (0.1 mM EDTA, pH 8) and a brief heating step. These differences can leave varying amounts of residual magnesium ions in purified RNA, thereby affecting subsequent HPLC–SEC purification. At 30°C, MEGAclear-purified RNA displayed sharper chromatographic profiles than RNA purified with Zymo columns, and the improvement was more pronounced for the PIE construct than for T4lig2 (Supplementary Fig. S8). Applying MEGAclear elution conditions to Zymo columns restored circRNA separation efficiency to MEGAclear levels (Supplementary Fig. S8, Condition 3), confirming the impact of magnesium ions on circRNA HPLC–SEC purification.

### HPLC–SEC purifies circRNAs from crude enzymatic reactions

**Figure 6.**
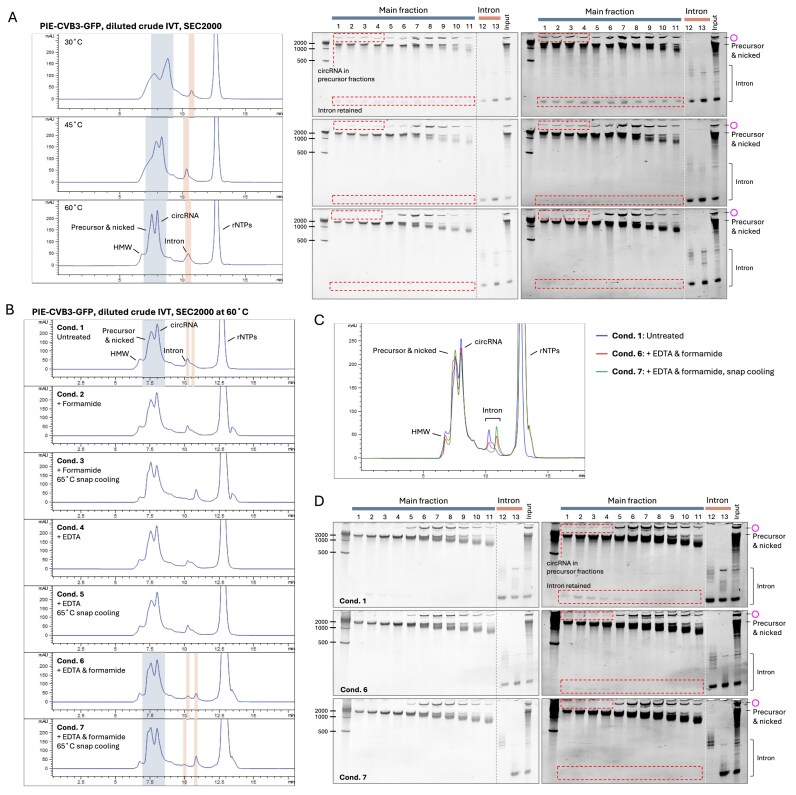
HPLC–SEC purification of PIE–CVB3–GFP circRNAs without prior RNA cleanup. (**A**) HPLC–SEC purification of unpurified PIE–CVB3–GFP IVT products at different column temperatures. Unpurified PIE–CVB3–GFP IVT products (20 µl) were treated with DNase I, circularized by incubation with 2.5 µl 10× T4 ligase buffer (NEB) and 1 µl GTP (100 mM) in 50 µl at 55°C for 30 min, and diluted to 100 µl with MEGAclear elution buffer (0.1 mM EDTA, pH 8.0). A 2 µl aliquot was adjusted to 10 µl with the MEGAclear elution buffer and subjected to HPLC–SEC purification using the SEC-2000 column at 30°C, 45°C, or 60°C. The main and spliced-out intron fractions, shown in blue and red, respectively, were analyzed by 3.5% urea PAGE. Gels were visualized using low- and high-contrast settings (left and right images, respectively). (**B**) Effect of sample pretreatment on HPLC–SEC purification. Following the same sample preparation steps as in panel (A), samples were pretreated using the indicated conditions before injecting them into the SEC-2000, column heated to 60°C. The following pretreatment conditions were conducted: Condition 1: no additives. Condition 2: 50% formamide. Condition 3: same as Condition 2 but with heating and snap cooling (65°C for 3 min, then on ice). Condition 4: same as Condition 2 but adjusted to 10 mM EDTA. Condition 5: same as Condition 4 but followed by heating and snap cooling (as earlier). Condition 6: adjusted to 10 mM EDTA and 50% formamide. Condition 7: Same as Condition 6 but with heating and snap cooling. (**C**) Overlaid chromatogram of Conditions 1, 6, and 7 from panel (B). (**D**) Samples of Conditions 1, 6, and 7 were analyzed by 3.5% urea PAGE, using low and high contrast settings (left and right panels, respectively).

We explored whether the RNA cleanup step prior to HPLC–SEC purification could be omitted. Based on the sample pretreatment tests above, we reasoned that the magnesium ions present from IVT and circularization reaction hindered circRNA separation in SEC. Consistent with our expectations, the circRNAs from both the PIE and STS constructs exhibited poor separation at a column temperature of 30°C (Fig. 6A and Supplementary Fig. S9A). The chromatographic profiles of unpurified PIE and STS IVT products nearly mirrored those of spin column-purified RNA samples treated with MgCl_2_ and snap cooling (Fig. 5B, Supplementary Fig. S7, Condition 4), where introns and circRNAs co-fractionated with precursors. Raising the column temperature to 45°C enhanced separation, with circRNAs and spliced-out introns appearing in later peaks. At 60°C, the separation resolution improved further, closely resembling spin column-purified RNA samples, in which only minimal intron retention was detected in the circRNA fractions (Fig. 6A and Supplementary Fig. S9A). To remove the remaining trace of retained introns in PIE–CVB3–GFP circRNA fractions, we combined the strategies of snap cooling and the addition of EDTA and/or formamide (Fig. 6B). With 10 mM EDTA and 50% formamide present in the samples before injection, the intron content in the circRNA fractions was significantly reduced, and snap cooling further eliminated intron retention to an undetectable level, even in the overexposed PAGE gels (Fig. 6D). Additionally, we observed an increased signal of the nicked RNA peak in PIE samples treated with EDTA, formamide, and snap cooling (Fig. 6B and C). The high-molecular-weight (HMW) aggregates were dissolved in the presence of EDTA/formamide and heating conditions, leading to an increased nicked circRNA signal. Meanwhile, removing introns also contributed to the decreased UV signals in circRNA fractions. However, this synergistic benefit of formamide/EDTA and snap cooling for circRNA separation was not observed in the STS construct, where all intron products were already removed from both precursor and circRNA fractions at a column temperature of 60°C (Supplementary Fig. S9A).

**Figure 7.**
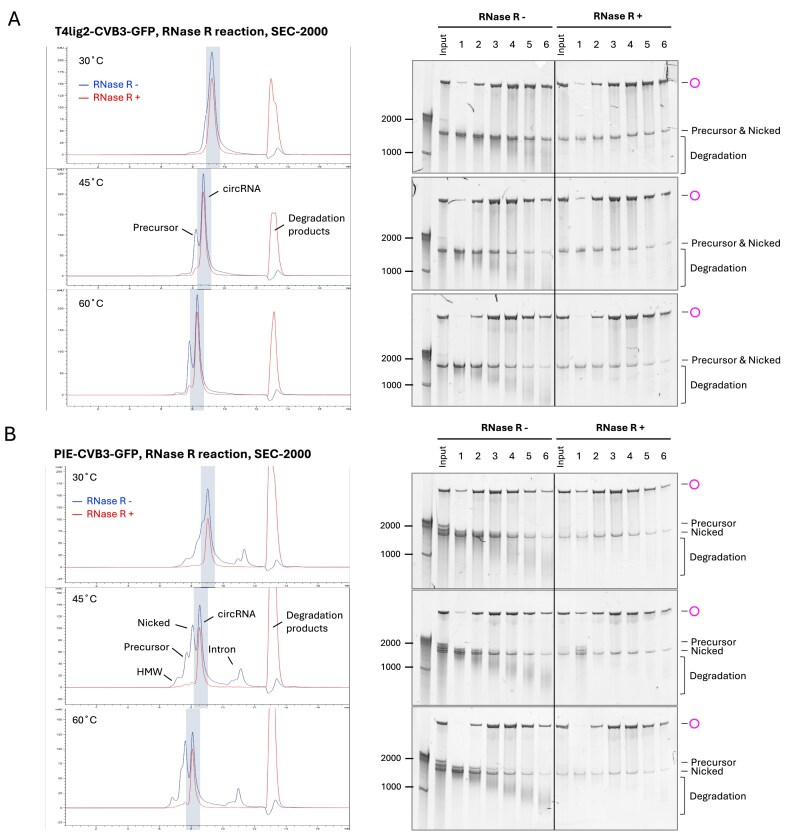
HPLC–SEC analysis of unpurified RNase R reactions. T4lig2–CVB3–GFP (**A**) and PIE–CVB3–GFP (**B**) RNAs were analyzed using SEC-2000 at different temperatures after RNase R treatment without spin column purification. Chromatographic profiles of RNase R-treated (red) and untreated (blue) samples are overlaid. Collected HPLC fractions (shaded in blue) were analyzed by 3.5% urea PAGE.

One possible explanation is that PIE samples were supplemented with additional magnesium ions, followed by a heating process at 55°C for 15 to 30 min to improve the yield of the circularization, which may require harsher denaturing conditions to separate circRNAs from intron products. In contrast, no extra Mg^2+^ or heating step was introduced for STS constructs after IVT. Additionally, the sequence difference between the group I intron species and the construct design could also affect the intermolecular binding affinity between the excised intron and the circRNA (Fig. 1B).

After establishing HPLC–SEC purification for crude IVT products, we next assessed whether this translates to other enzymatic reactions and tested unpurified RNase R-treated samples (Fig. 7 and Supplementary Fig. S10). Elevated column temperatures also improved circRNA separation from unpurified RNase R-treated samples, with markedly better resolution at 45°C and 60°C than at 30°C. Because no cleanup was performed, RNase R-treated and untreated samples could be directly compared by HPLC–SEC without RNA loss from spin columns. RNase R selectively digested degraded RNA that would otherwise co-elute with circRNA in HPLC-SEC, producing a sharper peak with less tailing. Together, these results show that HPLC–SEC complements, rather than replaces, exonuclease treatment for circRNA purification. Moreover, RNase R and HPLC–SEC act synergistically: RNase R removes linear contaminants, while HPLC–SEC further resolves circRNAs from remaining species, including exonuclease-resisted linear RNAs and high molecular weight aggregates.

### RNA crosslinking disrupts circRNA separation

**Figure 8.**
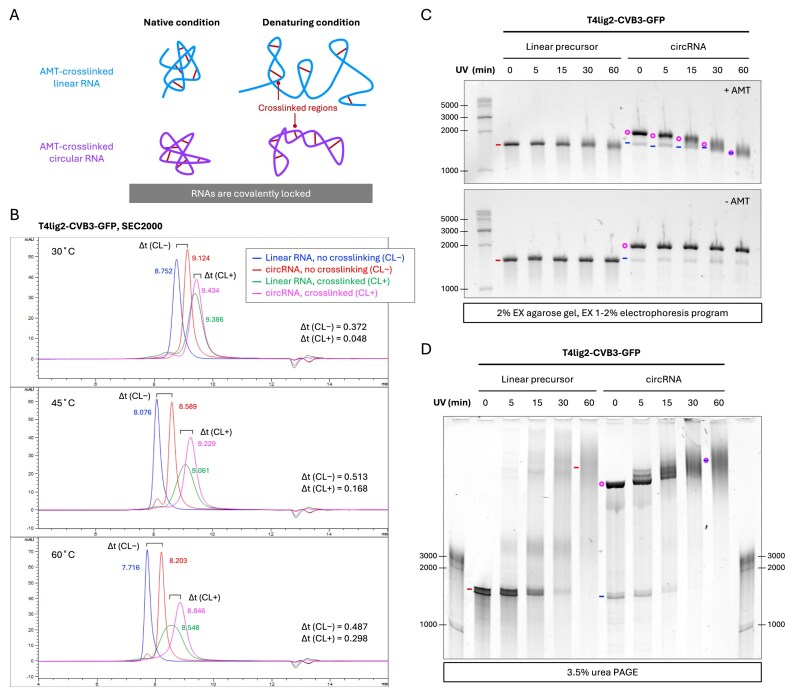
Effect of crosslinking on circRNA separation. (**A**) Schematic illustration of AMT-crosslinked linear and circular RNAs under native and denaturing conditions. (**B**) HPLC–SEC analysis of crosslinked RNA. RNase R-enriched T4lig2–CVB3–GFP circRNA and HPLC–SEC-purified linear precursor were crosslinked by 60 min of UV irradiation with or without AMT, followed by column purification prior to HPLC–SEC. Retention times are color-coded for each sample, and Δt denotes the difference in retention time between linear and circular RNAs under crosslinked (CL+) and non-crosslinked (CL–) conditions. (**C**) E-Gel analysis of crosslinked RNA. T4lig2–CVB3–GFP precursor and circRNA were UV irradiated for the indicated times with AMT in the presence (+AMT) or absence (–AMT, non-crosslinked control). Samples were analyzed by 2% EX gels (EX 1%–2% program, 20 min). (**D**) Crosslinked RNAs from the same reactions from (C) were analyzed by 3.5% urea PAGE. Symbols indicate annotated bands: circRNA (magenta circle), precursor (red line), and nicked circRNA (blue line).

To further demonstrate that RNA denaturation is essential in circRNA separation, we tested the effect of crosslinking on T4lig2–CVB3–GFP circRNA and precursor prior to purification. Upon 365 nm UV exposure, 4′-amino-methyl trioxsalen hydrochloride (AMT), a widely used RNA crosslinker, intercalates into RNA duplexes and forms photocycloaddition between pyrimidines, producing covalent crosslinks [50]. These crosslinks lock RNA duplexes intramolecularly, thereby preventing denaturation-induced conformational changes (Fig. 8A). On 2% EX agarose gels, crosslinking increased the electrophoretic mobility of T4lig2–CVB3–GFP RNAs (Fig. 8C, upper panel), with circRNAs showing larger band shifts than linear precursors. In contrast, in 3.5% urea PAGE, crosslinking reduced the mobility of both species, with the effect markedly stronger for linear RNA (Fig. 8D). Despite these opposing mobility changes, circRNA and precursor bands overlapped after 60 min of crosslinking in both gel systems, presumably reflecting multiple crosslinking events that made linear and circular RNAs structurally indistinguishable. UV irradiation alone (–AMT), used as a non-crosslinking control, caused no changes (Fig. 8C, lower panel).

Next, we analyzed crosslinked T4lig2–CVB3–GFP RNAs by HPLC–SEC (Fig. 8B). At 30°C, the fractions of crosslinked circRNA and linear precursor completely overlapped. At higher temperatures they became partially resolved, but the retention time difference (Δt) remained much smaller than for non-crosslinked RNAs. Interestingly, the peak of crosslinked precursor broadened with increasing column temperature (Fig. 8B). This likely reflects the fact that linear RNAs, being less structurally constrained than circular ones, adopt greater conformational heterogeneity upon crosslinking, which is further amplified at elevated temperatures.

Overall, RNA crosslinking impaired circRNA separation in both gel electrophoresis and HPLC–SEC, underscoring the critical role of RNA denaturation in enabling circRNA separation.

## Discussion

In this study, we investigated the fundamental principle for separating *in vitro*-synthesized circRNAs from linear side-products generated from three synthesis methods. We demonstrated that the denaturation condition is crucial for effective circRNA separation in both gel electrophoresis and HPLC size exclusion chromatography.

**Figure 9.**
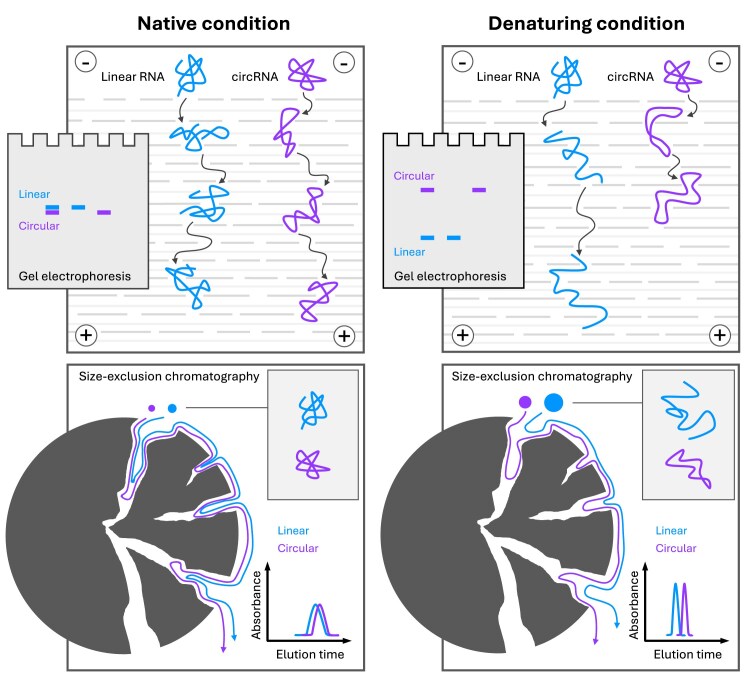
Mechanism of circRNA separation.

Under native conditions, RNA molecules adopt complex structures. During gel electrophoresis, the negatively charged RNA molecules are propelled through a gel matrix by the electric field. RNA molecules of different shapes and hydrodynamic sizes experience varying degrees of drag and retention as they thread through the gel pores, leading to differences in their electrophoretic mobility. Non-denatured linear and circular RNAs of identical sequences are similar in hydrodynamic size, making them difficult to separate in native gels. However, circRNAs probably can form overall more compact structures due to their circular topology, allowing them to migrate slightly faster than their linear counterparts during electrophoresis. In size exclusion chromatography, the migration of RNA molecules is driven by pressure-induced liquid flow. Here, the difference in migration speed is primarily determined by the average hydrodynamic size of RNA molecules. Large molecules bypass the porous stationary phase, traveling fast, while small molecules enter the pores more frequently, leading to delayed elution [29]. Like native gel electrophoresis, in SEC, linear RNAs presumably form less compact structures exhibiting larger average hydrodynamic sizes compared to circRNAs. Consequently, linear RNAs are eluted earlier than their circular counterparts. However , such difference in average hydrodynamic sizes induced by circular topology appears to be inadequate for effective circRNA separation under native conditions (Fig. 9, left panel).

Under denaturing conditions, such as high temperature or chemical denaturants like urea and formamide, the complex structures of RNA molecules are unfolded, induced by the disruption of hydrogen bonds. During denaturing gel electrophoresis, linear RNAs are likely forced into an extended single-stranded conformation, allowing them to navigate the gel matrix by “snaking” through the pores. In contrast, circRNAs still stay in a looped structure, resulting in increased friction as they may traverse the gel pores in a manner resembling unpaired double-stranded RNA molecules. This leads to markedly slower migration and distinct banding patterns compared to their linear counterparts. Considering the agarose E-Gel system, for which the gel components and the electrophoresis parameters are proprietary and undisclosed, we assume that the excessive heat buildup during electrophoresis is denaturing the RNA, consequently facilitating circRNA separation. In size exclusion chromatography, under denaturing conditions, both linear and circular RNAs become “floppier” and more expanded than their native conformations, leading to larger average hydrodynamic sizes and generally shorter elution times. Importantly, due to the structural constraints of a circular topology, even when fully denatured, circRNAs can only reach half the maximum length of their stretched linear counterparts. This results in a greater difference in average hydrodynamic sizes between circular and linear RNAs under denaturing conditions, thus improving the efficiency of circRNA separation (Fig. 9, right panel). We speculate this principle could extend to CGE for effective circRNA separation by using only formamide as the capillary gel solvent, a method successfully applied in high-resolution linear mRNA analysis [51].

Besides the structural status of RNA molecules, the size of circRNA directly impacts its separation in gel electrophoresis. Our investigation primarily focused on the CVB3–GFP circRNAs of ~1500 nt, while circRNAs of varying sizes can exhibit distinct electrophoretic patterns. For instance, small RNA circles (10 to 25 nt) migrate faster than their linear counterparts in 12% to 18% denaturing urea PAGE [46, 52, 53]. The sieving ability of the gel matrix is also strongly influenced by the concentration of agarose or polyacrylamide. Our study revealed that circular and linear CVB3–GFP RNAs of equivalent lengths overlapped in 1% AGE, regardless of whether self-cast gels or commercial E-Gels were used. This overlap likely stems from the pore size of the gel matrix being too large, preventing linear RNA molecules from adopting a single-stranded conformation as they migrate through the pores, thereby reducing the separation efficiency for circRNAs. Notably, a recent study showed that 0.8% native agarose gel with extended electrophoresis time successfully separated a super-large RNA circle of 8600 nt from its linear counterpart [10]. These findings highlight that the electrophoretic separation of circRNAs depends on the interplay between RNA size and the sieving effect of the gel matrix.

An intriguing observation in our study is that using HPLC–SEC at 45°C with a Tris-EDTA mobile phase improved the separation of T4-ligated and PIE-generated circRNAs at low pH (<4.5) and neutral pH (7.5), whereas intermediate pH levels (5.5–6.5) showed poorer performance. One possible explanation for better circRNA separation at low pH is that protonation at N3 position in cytosine (pKa 4.2) [54] disrupts the hydrogen bonds in G-C pairs and promotes denaturation of the RNA molecules. Thus, both linear and circular RNAs may become less structured and more homogenous, allowing them to be separated more effectively in HPLC–SEC.

Magnesium ions in RNA samples are an easily overlooked factor because they can co-purify with the RNA. Even a small amount can significantly affect HPLC–SEC performance, leading to intron retention in PIE and STS circRNA fractions. Intron co-elution in circRNA fraction is likely caused by the remaining base pairing of the intronic ribozyme sequences to the guide sequences of the exons (Fig. 1B). We found that column heating, combined with the addition of EDTA to chelate magnesium ions and formamide to destabilize hydrogen bonding in the RNA samples, following snap cooling to avoid intermolecular base pairing, effectively resolved this issue.

After our extensive efforts to optimize circRNA separation using HPLC–SEC, several limitations remain. (i) Even at optimized conditions, SEC purification cannot fully separate circRNA from smaller side products. Since circRNAs are eluted later than their linear counterparts, circRNA can co-elute with impurities from *in vitro* transcription or degradation products during HPLC processing (Fig. 7 and Supplementary Fig. S10). As a result, additional purification steps, including RNase R treatment, are required to increase the purity of circRNAs. This issue is further exacerbated when working with smaller circRNAs, as their fractions may overlap with the spliced-out intron fragments that have similar elution times. (ii) The separation efficiency of circRNAs in SEC is further compromised when injecting larger amounts of RNAs, which hampers upscaling purification (Supplementary Fig. S11). (iii) RNA is susceptible to degradation at high temperatures and shear force exerted by the column matrix, leading to increased nicking of circRNAs. However, according to chromatographic profiles, we did not observe significant loss of circRNA signal when heating the column, likely attributed to the short processing time, typically around 10 min from injection to elution in SEC-2000. (iv) Heating the column is discouraged by the manufacturer, as excessive heat can shorten its lifespan and diminish its performance. Clearly, further development of scalable circRNA separation methodologies is still needed.

Already in the 1970s, scientists used denaturing PAGE to successfully separate and analyze *in vitro*-synthesized circRNAs [19, 52]. This pioneering achievement provided a powerful and straightforward biochemical approach for studying circRNAs, which are commonly associated with self-splicing introns—a prominent life science topic during the 1980s. Research on synthesized circRNAs fell into abeyance around the turn of the 21st century but resurged with the advent of mRNA vaccines. However, the current rapid development of circRNA research has been occasionally accompanied by a diminished focus on fundamental RNA biology knowledge. In our study, we revisit previously established concepts and further explore the principle underlying circRNA separation. We believe our findings will advance the development of circRNA-based biotechnology and therapeutics, paving the way for innovative treatment strategies in the future.

## Supplementary Material

gkaf1160_Supplemental_File

## Data Availability

Data supporting the findings of this study are available within the article and its supplementary data.
