# Supplementary material for: RNA denaturation underlies circular RNA separation
Source: Nucleic Acids Res. 2025 Nov 20;53(21):gkaf1160. doi: 10.1093/nar/gkaf1160 (PMC12630139; doi:10.1093/nar/gkaf1160)
Supplement: gkaf1160_Supplemental_File [file gkaf1160_supplemental_file.pdf]

Supplementary Materials for

RNA denaturation underlies circular RNA separation

Yanyi Jiang, Jørgen Kjems*

* Corresponding author. Email: jk@mbg.au.dk

This PDF file includes:

Figure S1. Validation of RNA circularity
Figure S2. HPLC-SEC column setup and purification of STSΔP10-CVB3-GFP using SEC-1000 or SEC-2000
Figure S3. HPLC-SEC purification of circRNAs using SEC-4000
Figure S4. HPLC-SEC purification of T4lig2-origami using SEC-1000 and SEC-2000 columns
Figure S5. HPLC-SEC purification of ribozymatically synthesized circRNAs using mobile phases of the indicated pH
Figure S6. HPLC-SEC purification of ribozymatically synthesized circRNAs with mobile phases containing the indicated sodium chloride concentrations
Figure S7. HPLC-SEC purification of STSΔP10-CVB3-GFP RNA with the indicated sample pre-treatment conditions
Figure S8. Elution conditions affect circRNA separation in HPLC-SEC
Figure S9. HPLC-SEC purifies STSΔP10-CVB3-GFP circRNAs without prior RNA cleanup
Figure S10. HPLC-SEC analysis of unpurified RNase R reactions of STSΔP10-CVB3-GFP
Figure S11. RNA loading capacity of SEC-2000 column
Figure S12. Unprocessed gel images from Figure 2
Figure S13. Unprocessed gel images from Figure 3

Table S1. The sequence of transcribed precursors
Table S2. The sequence of DNA probes for RNase H assay

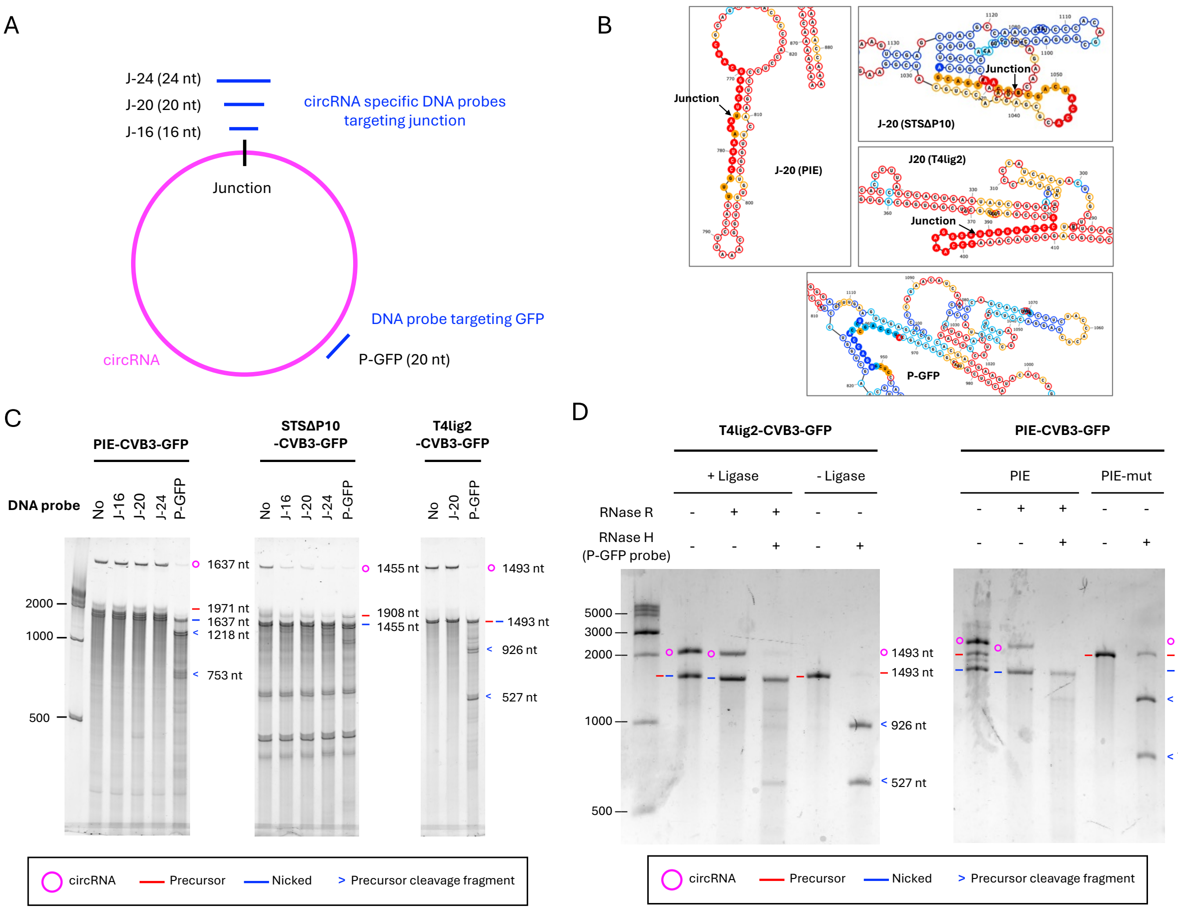

Figure S1. Validation of RNA circularity. (A) Schematic of RNase H probe binding sites. **(B)** RNAFold predictions of probe-binding regions. RNA sequences complementary to the DNA probes are highlighted with solid circles, and circRNA junctions within the probe-binding regions are indicated by black arrows. **(C)** RNase H assay for circRNA validation. RNA samples from three constructs were treated with RNase H using the denoted DNA probes presented in (A) and analyzed by 3.5% urea PAGE. **(D)** Combined RNase H and RNase R assays. Products produced from circRNAs (+ligase, PIE) and their linear precursors (–ligase, PIE-mut) upon combining RNase R and RNase H treatments were analyzed by 2% EX agarose gel. Symbols indicating the gel bands are: circRNA (magenta circle), precursor (red line), nicked circRNA (blue line), precursor cleavage product (blue arrow).

A

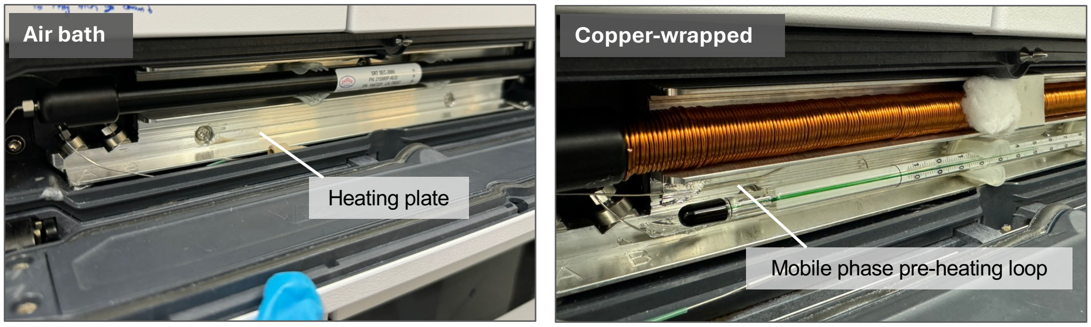

Setting (˚C)	30.0	45.0	60.0
Oven air bath (˚C)	28.5 - 29.0	36.5 - 40.5	48.0 - 53.0

B

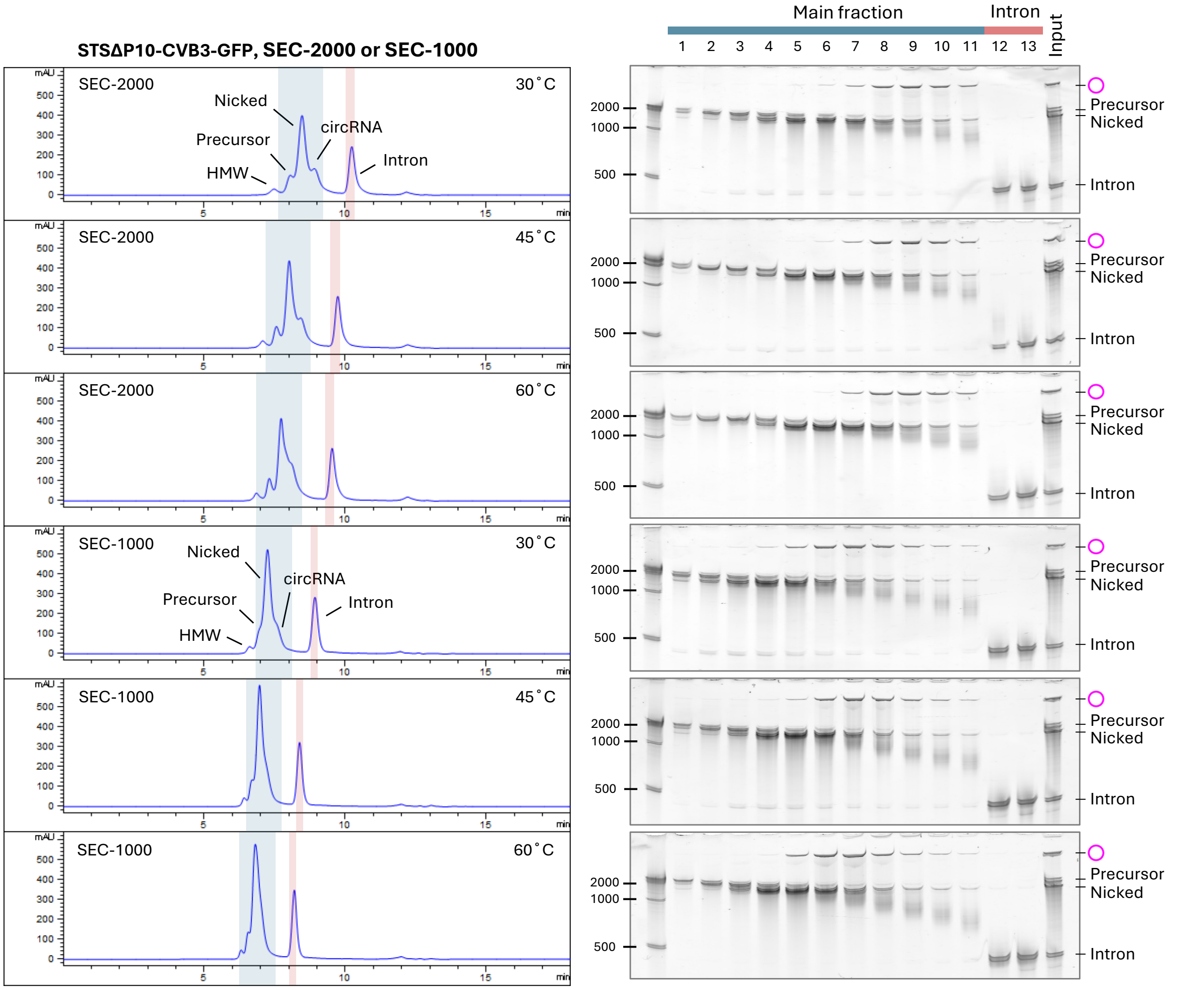

Figure S2. HPLC-SEC column setup and purification of STSΔP10-CVB3-GFP using SEC-1000 or SEC-2000. (A) The HPLC-SEC column setup. The air temperatures in the column oven were measured and compared to the setting temperatures. **(B)** circRNA separation of STSΔP10-CVB3-GFP in SEC-1000/2000 with column heating. RNAs were purified with HPLC-SEC at 30, 45 and 60˚C. The eluted fractions were analyzed using 3.5% urea PAGE, with main and intron fractions marked in blue and red.

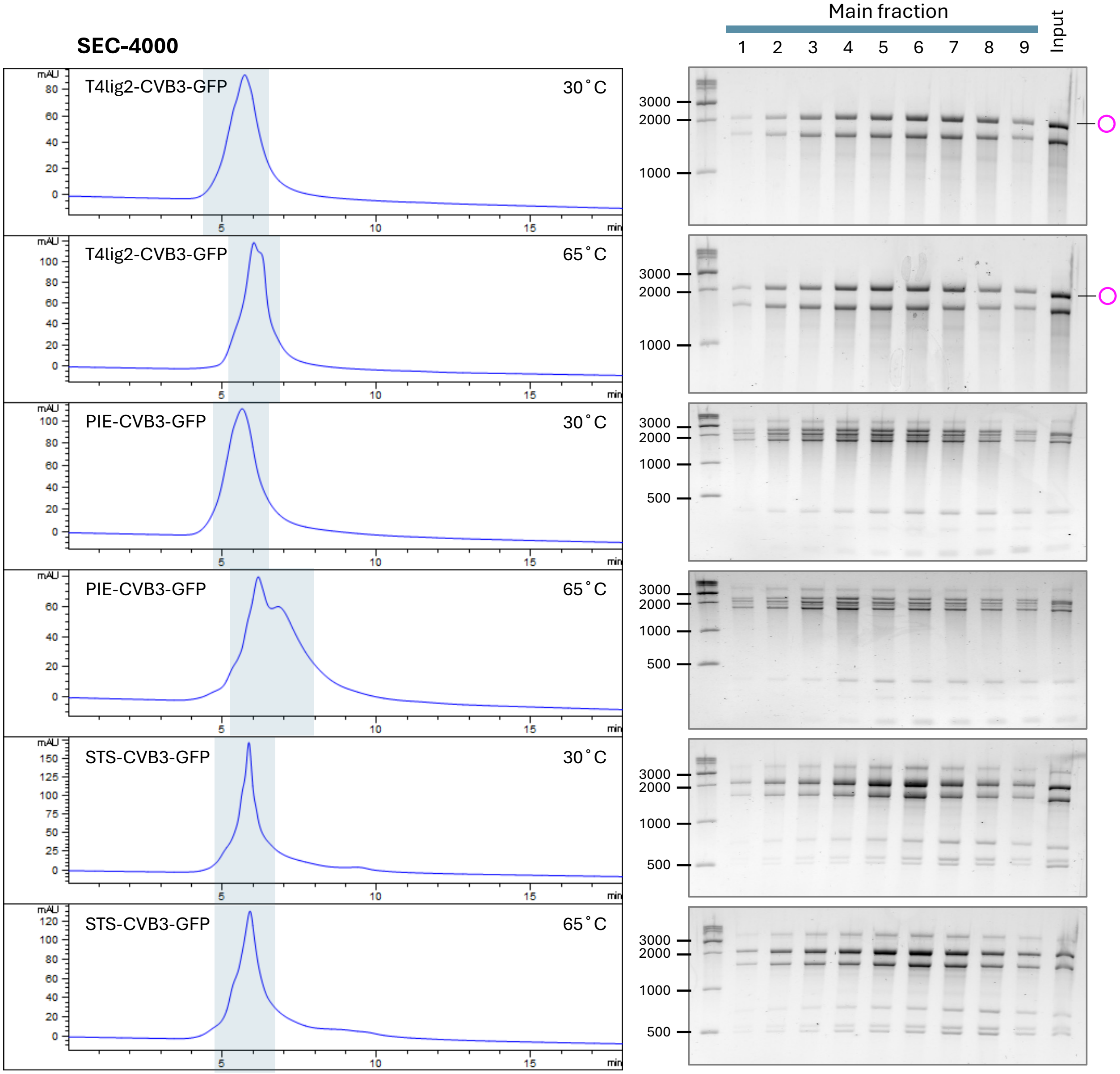

Figure S3. HPLC-SEC purification of circRNAs using SEC-4000. Cleaned-up RNA samples were injected into the SEC-4000 column at 30 or 65˚C. The collected fractions (depicted in blue areas) were analyzed with 2% EX gel electrophoresis. The circRNAs are marked with magenta circles to the right.

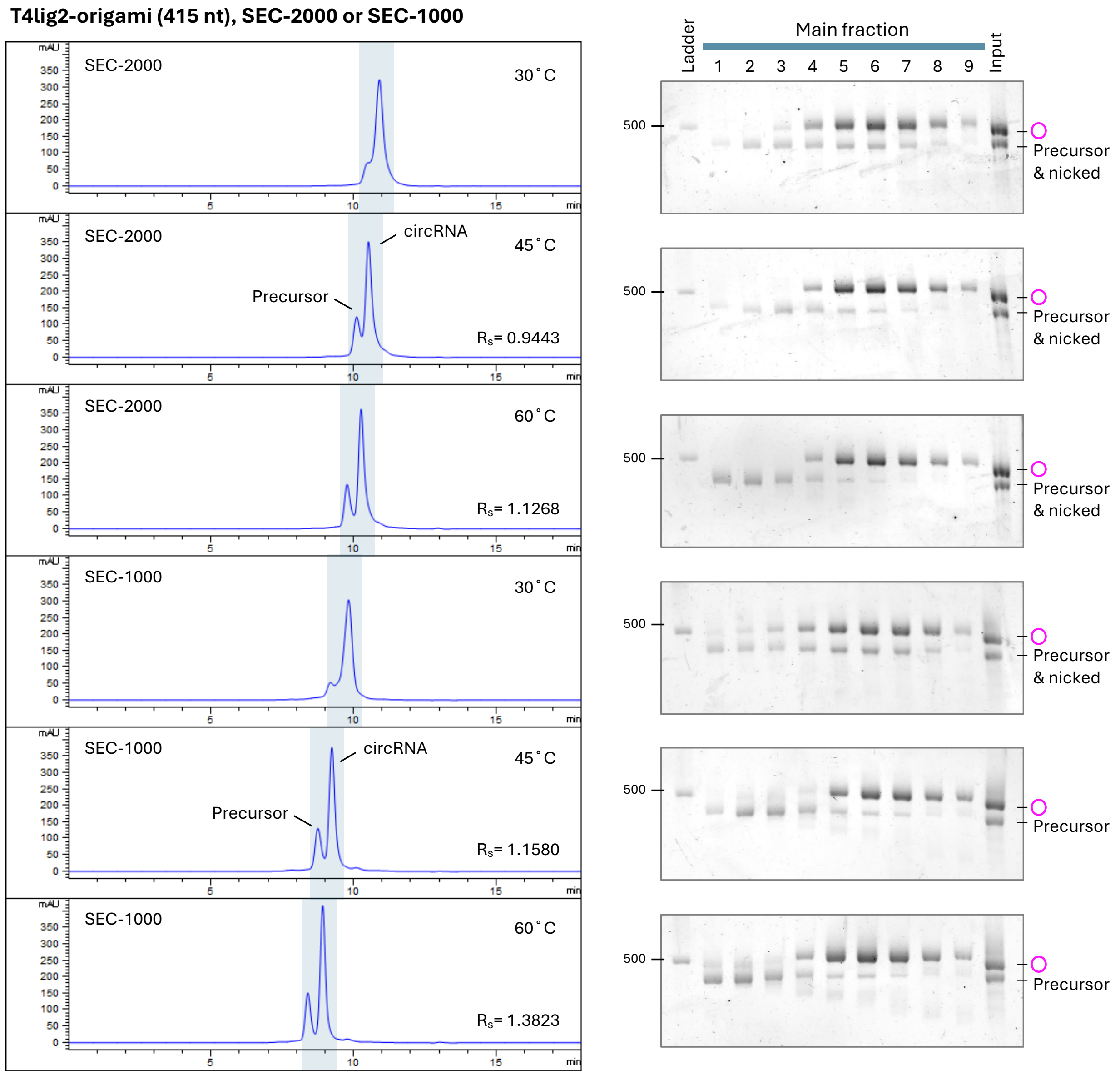

Figure S4. HPLC-SEC purification of T4lig2-origami using SEC-1000 and SEC-2000 columns. circRNA separation was assessed at different column temperatures. Fractions indicated in blue were analyzed by 2% EX gels.

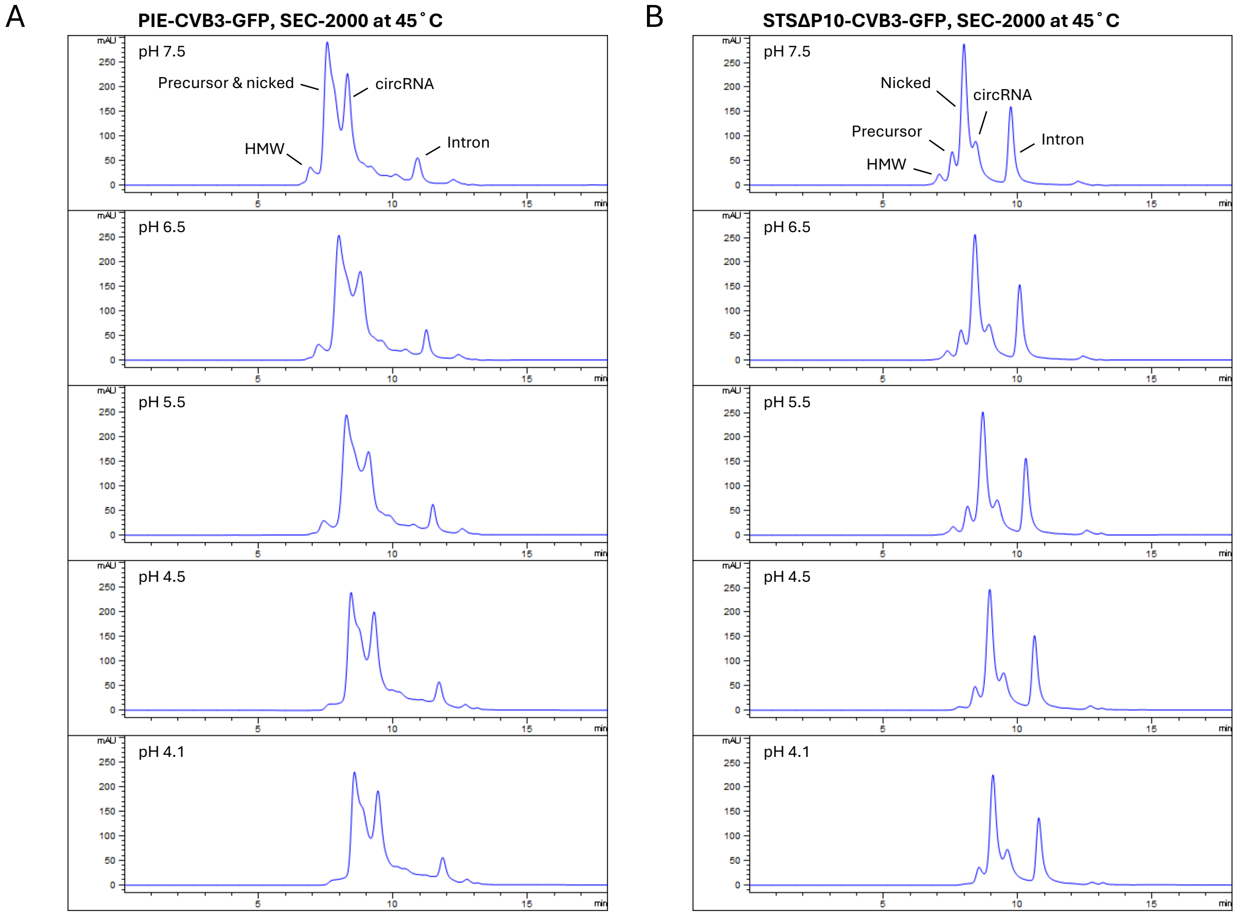

Figure S5. HPLC-SEC purification of ribozymatically synthesized circRNAs using mobile phases of the indicated pH. (A) PIE- and **(B)** STSΔP10-CVB3-GFP RNA samples were purified by the SEC-2000 column at 45˚C using mobile phases with varying pH values.

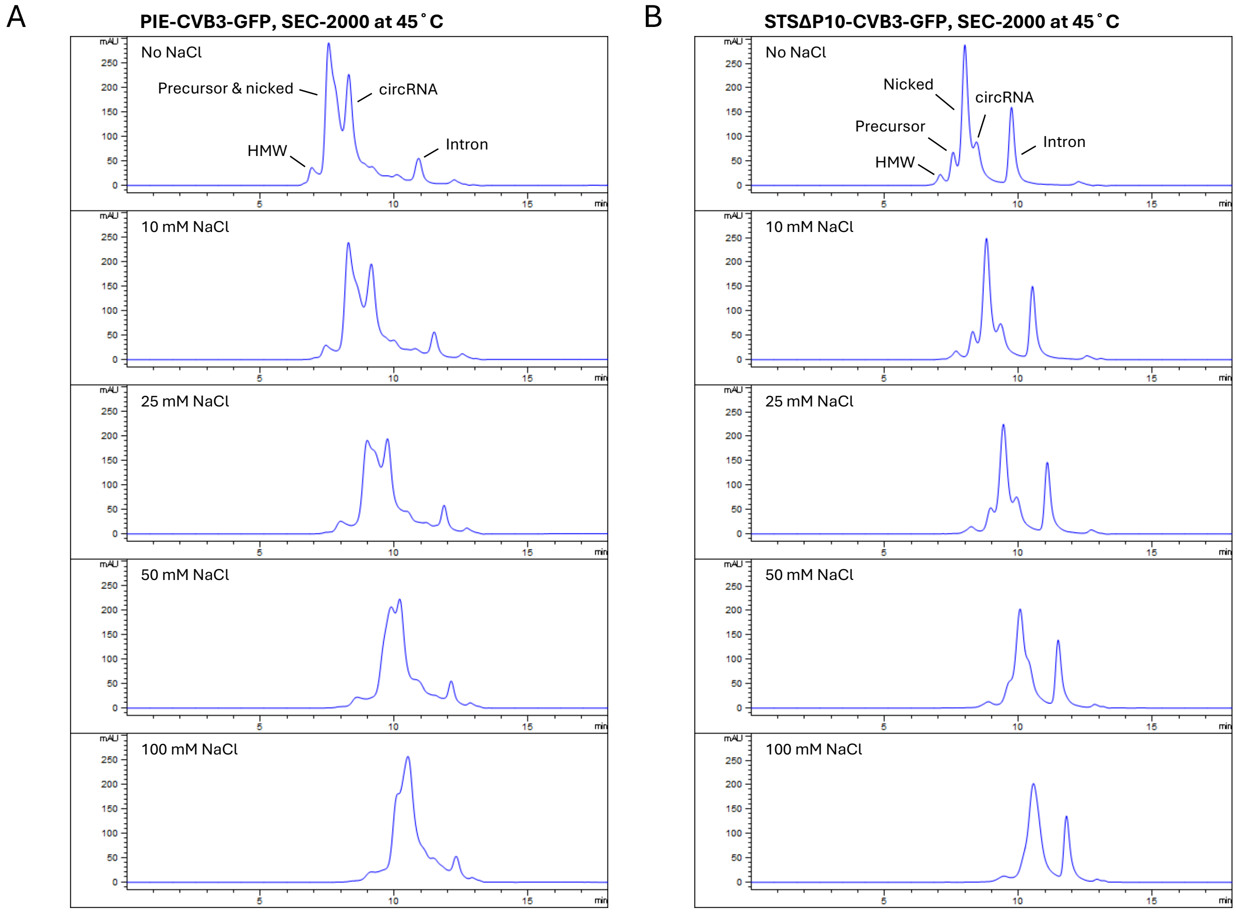

Figure S6. HPLC-SEC purification of ribozymatically synthesized circRNAs with mobile phases containing the indicated sodium chloride concentrations. (A) PIE- and **(B)** STSΔP10-CVB3-GFP RNA samples were purified by the SEC-2000 column at 45˚C with mobile phases of different salt concentrations.

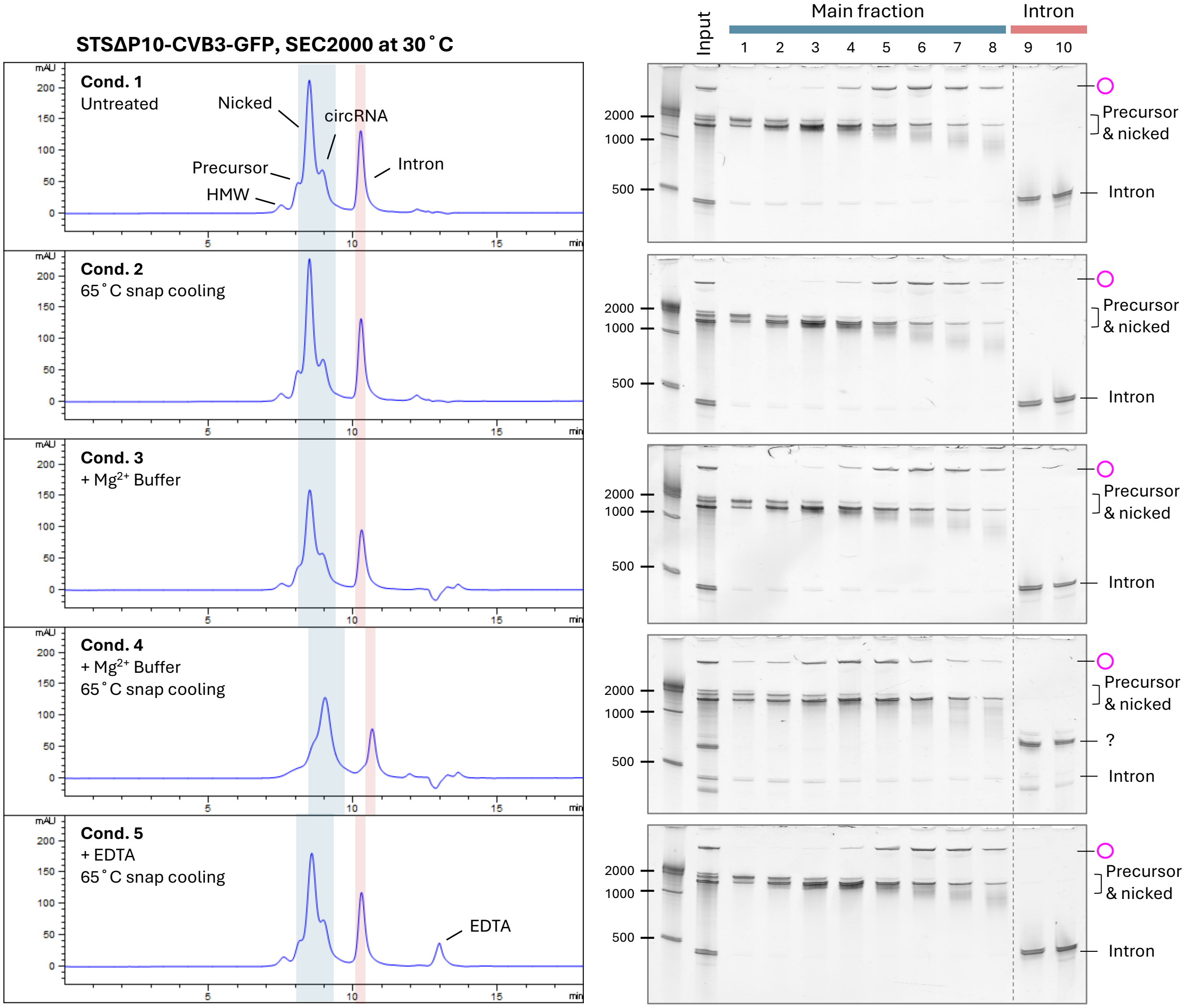

Figure S7. HPLC-SEC purification of STSΔP10-CVB3-GFP RNA with the indicated sample pre-treatment conditions. Column-purified STSΔP10-CVB3-GFP RNA samples were processed as in Figure 5, except for the last condition. Condition 5: EDTA (10 mM) was added to the cleaned-up RNA, followed by snap cooling. HPLC-SEC fractions were analyzed by 3.5% urea PAGE. The analyzed main and intron fractions are shown in blue and red, respectively.

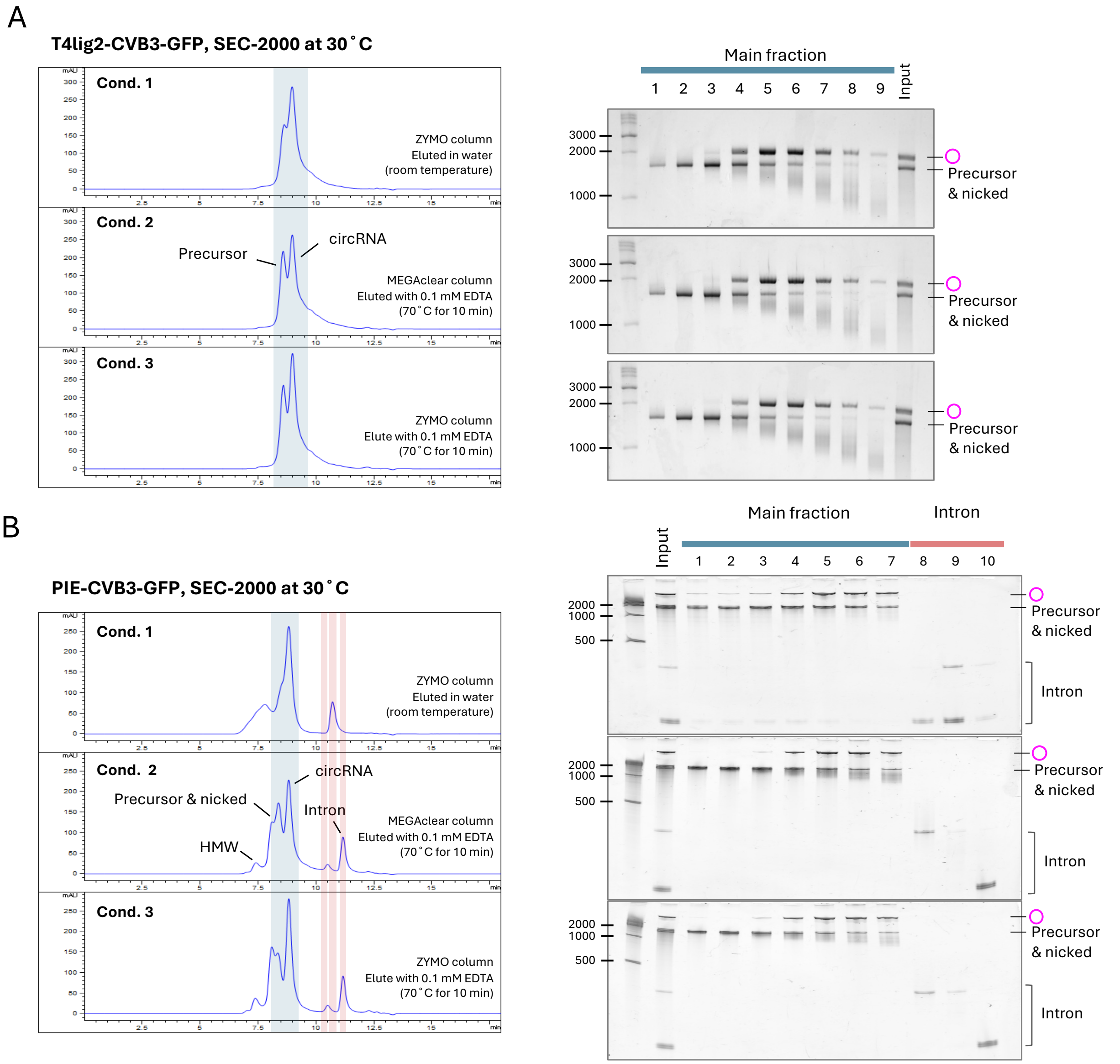

Figure S8. Elution conditions affect circRNA separation in HPLC-SEC. (A and B) Prior to HPLC injection, RNA was purified by spin columns under three conditions: (1) Zymo RNA Clean & Concentrator-25, eluted in water at room temperature; (2) Invitrogen MEGAclear, eluted in 0.1 mM EDTA (pH 8.0) after incubation at 70 °C for 10 min; (3) Zymo RNA Clean & Concentrator-25, eluted in 0.1 mM EDTA (pH 8.0) after incubation at 70 °C for 10 min. HPLC fractions of T4lig2-CVB3-GFP and PIE-CVB3-GFP were analyzed on 2% EX gels and 3.5% urea PAGE, with main and intron fractions highlighted in blue and red, respectively.

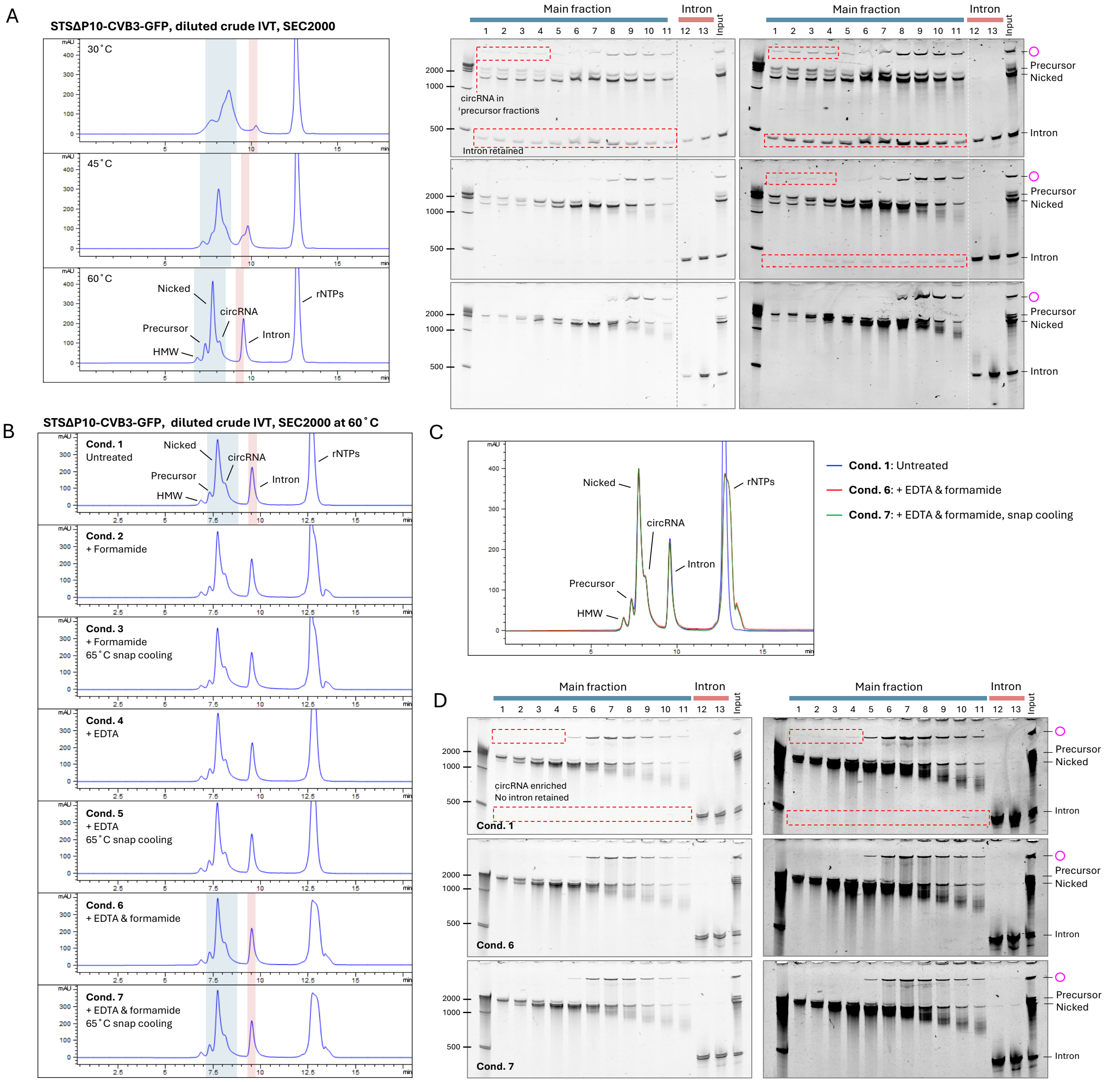

Figure S9. HPLC-SEC purifies STSΔP10-CVB3-GFP circRNAs without prior RNA cleanup. (A) HPLC-SEC purification of diluted crude STSΔP10-CVB3-GFP IVT products using the SEC-2000 column at 30˚C, 45˚C, and 60˚C. After IVT and DNase treatment, the crude product (50 µL) was diluted with MEGAclear elution buffer (0.1 mM EDTA, pH 8.0) to 100 µL. A 2 µL aliquot was further diluted to 10 µL using the same buffer, followed by HPLC-SEC purification. The main and intron fractions marked in blue and red, respectively, were analyzed by 3.5% urea PAGE. Gels were visualized using low and high contrast settings. **(B)** Pre-treatment of STSΔP10-CVB3-GFP IVT prior to HPLC-SEC, following the same procedure as in Figure 6B. The main and intron fractions, shown in blue and red, were analyzed by 3.5% urea PAGE. **(C)** Chromatogram overlay of the three pre-treatment sets from (B). **(D)** Samples of conditions 1, 6, and 7 from (B) were analyzed by 3.5% urea PAGE, visualizing gels using low and high contrast settings.

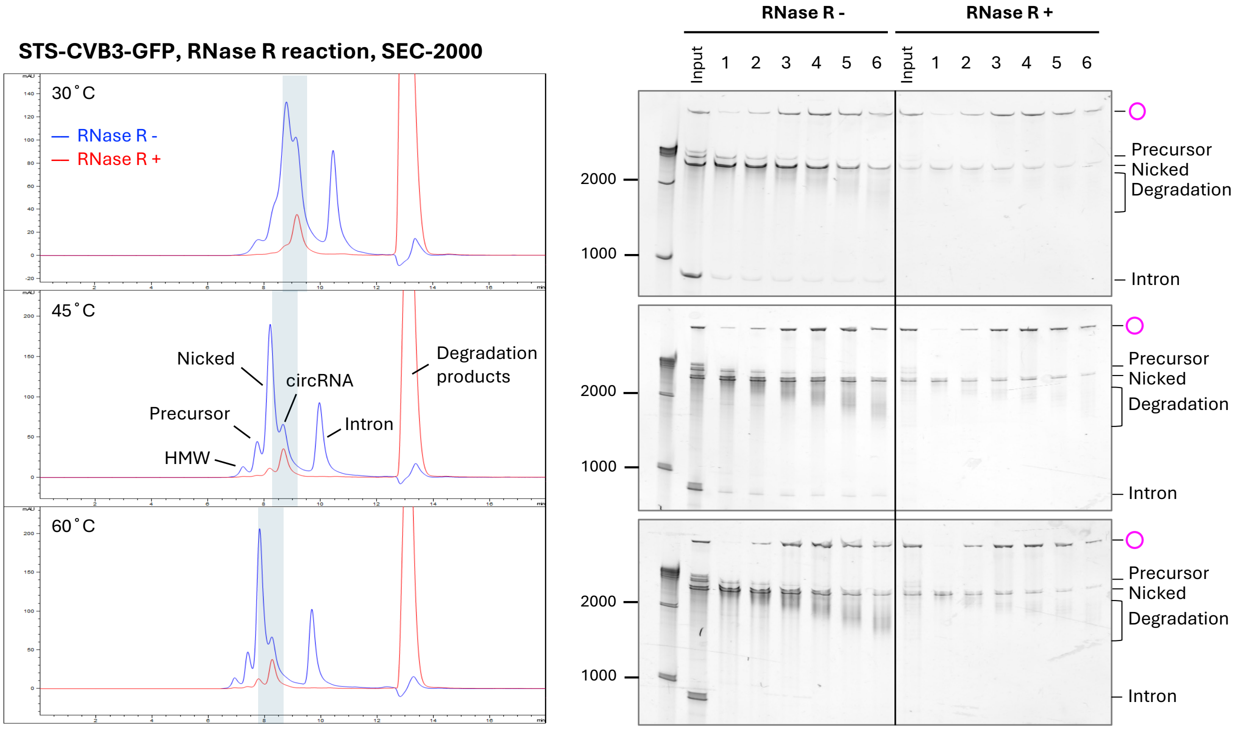

Figure S10. HPLC-SEC analysis of unpurified RNase R reactions of STSΔP10-CVB3-GFP. Samples were analyzed on SEC-2000 at different temperatures after RNase R treatment without spin column purification. Chromatographic profiles of RNase R treated (red) and untreated (blue) samples are overlaid. Collected HPLC fractions (shaded in blue) were analyzed by 3.5% urea PAGE are shown on the right.

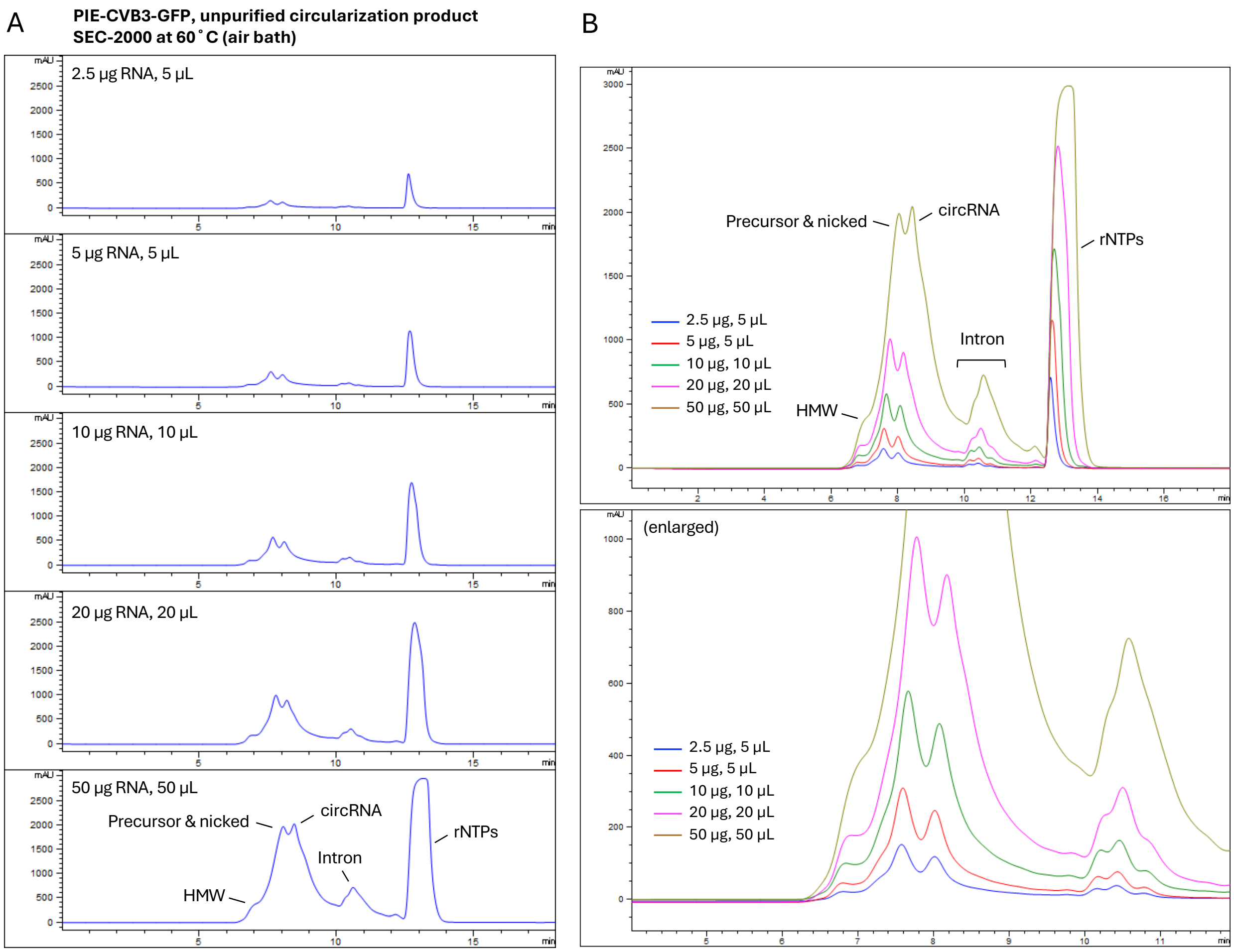

Figure S11. RNA loading capacity of SEC-2000 column. (A) HPLC-SEC purification of the PIE-CVB3-GFP unpurified circularization product using SEC-2000 column at 60˚C (air bath). After IVT and DNase treatment, the RNA product was purified by a spin column. Then, T4 RNA ligase buffer (NEB) and GTP (2 mM) were added, followed by a 15-minute incubation at 55˚C. The unpurified reaction was directly injected into HPLC-SEC with varying RNA amounts and injection volumes. **(B)** Chromatographic overlay shown in (A).

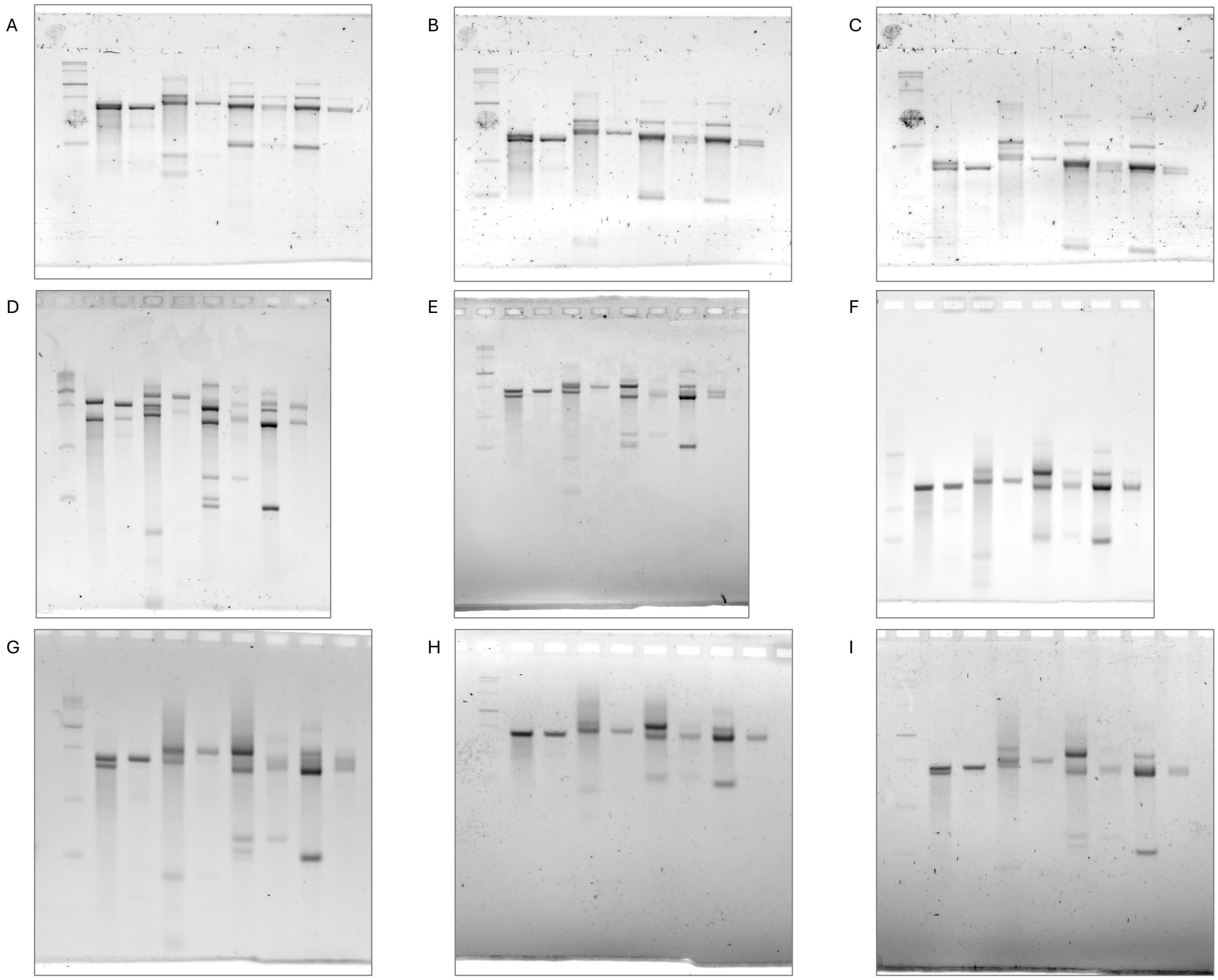

Figure S12. Unprocessed gel images from Figure 2. (A to C) Unprocessed gel images from Figure 2C. **(D to F)** Unprocessed images from Figure 2D. **(G to I)** Unprocessed images from Figure 2E.

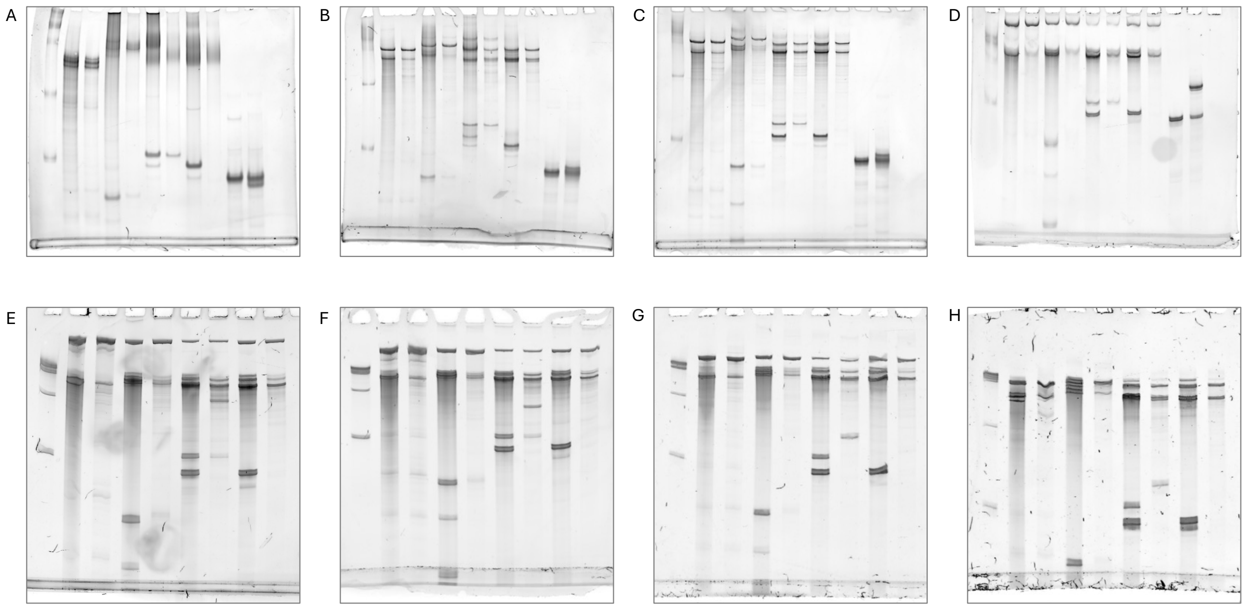

Figure S13. Unprocessed gel images from Figure 3. (A to D) Unprocessed gel images from Figure 3A. **(E to H)** Unprocessed gel images from Figure 3B.

Construct	Precursor Sequence (5' to 3')
**T4lig2-CVB3-GFP**	GGGGAAACCCAAACATGGGACGCTCTAATACAGACATGGTGCGAAGAGTCTATTGAGCTAGTTGGTAGT<br>CCTCCGGCCCCTGAATGCGGCTAATCCTAACTGCGGAGCACACACCCTCAAGCCAGAGGGCAGTGTGTC<br>GTAACGGGCAACTCTGCAGCGGAACCGACTACTTTGGGTGTCCGTGTTTCATTTTATTCCTATACTGGCT<br>GCTTATGGTGACAATTGAGAGATCGTTACCATATAGCTATTGGATTGGCCATCCGGTGACTAATAGAGCTA<br>TTATATATCCCTTTGTTGGGTTTATACCACTTAGCTTGAAAGAGGTTAAAACATTACAATTCATTGTTAAGT<br>TGAATACAGCAAAACTAGTGCCACCATGGTGAGCAAGGGCGAGGAGCTGTTCACCGGGGTGGTGCCCA<br>TCCTGGTCGAGCTGGACGGCGACGTAAACGGCCACAAGTTCAGCGTGTCTGGCGAGGGCGAGGGCGAT<br>GCCACCTACGGCAAGCTGACCCTGAAGTTCATCTGCACCACCGGCAAGCTGCCCGTGCCCTGGCCCACC<br>CTCGTGACCACCCTGACCTACGGCGTGCAGTGCTTCAGCCGCTACCCCGACCACATGAAGCAGCACGAC<br>TTCTTCAAGTCCGCCATGCCCGAAGGCTACGTCCAGGAGCGCACCATCTTCTTCAAGGACGACGGCAAC<br>TACAAGACCCGCGCCGAGGTGAAGTTCGAGGGCGACACCCTGGTGAACCGCATCGAGCTGAAGGGCAT<br>CGACTTCAAGGAGGACGGCAACATCCTGGGGCACAAGCTGGAGTACAACTACAACAGCCACAACGTCT<br>ATATCATGGCCGACAAGCAGAAGAACGGCATCAAGGCGAACTTCAAGATCCGCCACAACATCGAGGAC<br>GGCAGCGTGCAGCTCGCCGACCACTACCAGCAGAACACCCCCATCGGCGACGGCCCCGTGCTGCTGCC<br>CGACAACCACTACCTGAGCACCCAGTCCGCCCTGAGCAAAGACCCCAACGAGAAGCGCGATCACATGG<br>TCCTGCTGGAGTTCGTGACCGCCGCCGGGATCACTCTCGGCATGGACGAGCTGTACAAGTGATAATAGG<br>GGTTTTTAAAACAGCCTGTGGGTTGATCCCACCCACAGGCCCATTGGGCGCTAGCACTCTGGTATCACG<br>GTACCTTTGTGCGCCTGTTTTATACCCCCTCCCCCAACTGTAACTTAGAAGTAACACACACCGATCAACA<br>GTCAGCGTGGCACACCAGCCACGTTTTGATCAAGCACTTCTGTTACCCCGGACTGAGTATCAATAGACT<br>GCTCACGCGGTTGAAGGAGAAAGCGTTCGTTATCCGGCCAACTACTTCGAAAAACCTAGTAACACCGTG<br>GAAGTTGCAGAGTGTTTCGCTCAGCACTACCCCAGTGTAGATCAGGTCGATGAGTCACCGCATTCCCCA<br>CGGGCGACCGTGGCGGTGGCTGCGTTGGCGGCCTGCCCATGTTT
**T4lig2-PXT origami**	GGGGAAACCCAAACATGGGACGCTCTAATACAGACATGGTGCGAAGAGTCTATTGAGCTAGTTGGTAGT<br>CCTCCGGCCCCTGAATGCGGCTAATCCTAACTGCGGAGCACACACCCTCAAGCCAGAGGGCAGTGTGTC<br>GTAACGGGCAACTCTGCAGCGGAACCGACTACTTTGGGTGTCCGTGTTTCATTTTATTCCTATACTGGCT<br>GCTTATGGTGACAATTGAGAGATCGTTACCATATAGCTATTGGATTGGCCATCCGGTGACTAATAGAGCTA<br>TTATATATCCCTTTGTTGGGTTTATACCACTTAGCTTGAAAGAGGTTAAAACATTACAATTCATTGTTAAGT<br>TGAATACAGCAAAACTAGTGCCACCATGGGGGTGCACGAATGTCCTGCCTGGCTGTGGCTTCTCCTGTC<br>CCTGCTGTCGCTCCCTCTGGGCCTCCCAGTCCTGGGCGCCCCACCACGCCTCATCTGTGACAGCCGAGT<br>CCTGGAGAGGTACCTCTTGGAGGCCAAGGAGGCCGAGAATATCACGACGGGCTGTGCTGAACACTGCA<br>GCTTGAATGAGAATATCACTGTCCCAGACACCAAAGTTAATTTCTATGCCTGGAAGAGGATGGAGGTCG<br>GGCAGCAGGCCGTAGAAGTCTGGCAGGGCCTGGCCCTGCTGTCGGAAGCTGTCCTGCGGGGCCAGGCC<br>CTGTTGGTCAACTCTTCCCAGCCGTGGGAGCCCCTGCAGCTGCATGTGGATAAAGCCGTCAGTGGCCTT<br>CGCAGCCTCACCACTCTGCTTCGGGCTCTGGGAGCCCAGAAGGAAGCCATCTCCCCTCCAGATGCGGCC<br>TCAGCTGCTCCACTCCGAACAATCACTGCTGACACTTTCCGCAAACTCTTCCGAGTCTACTCCAATTTCC<br>TCCGGGGAAAGCTGAAGCTGTACACAGGGGAGGCCTGCAGGACAGGGGACAGATGATAATAGGGGTTT<br>TTAAAACAGCCTGTGGGTTGATCCCACCCACAGGCCCATTGGGCGCTAGCACTCTGGTATCACGGTACCT<br>TTGTGCGCCTGTTTTATACCCCCTCCCCCAACTGTAACTTAGAAGTAACACACACCGATCAACAGTCAGC<br>GTGGCACACCAGCCACGTTTTGATCAAGCACTTCTGTTACCCCGGACTGAGTATCAATAGACTGCTCACG<br>CGGTTGAAGGAGAAAGCGTTCGTTATCCGGCCAACTACTTCGAAAAACCTAGTAACACCGTGGAAGTTG<br>CAGAGTGTTTCGCTCAGCACTACCCCAGTGTAGATCAGGTCGATGAGTCACCGCATTCCCCACGGGCGA<br>CCGTGGCGGTGGCTGCGTTGGCGGCCTGCCCATGTTT
**PIE-CVB3-GFP**	GGGGGAGACCCTCGACCGTCGATTGTCCACTGGTCAACAATAGATGACTTACAACTAATCGGAAGGTGC<br>AGAGACTCGACGGGAGCTACCCTAACGTCAAGACGAGGGTAAAGAGAGAGTCCAATTCTCAAAGCCAA<br>TAGGCAGTAGCGAAAGCTGCAAGAGAATGAAAATCCGTTGACCTTAAACGGTCGTGTGGGTTCAAGTC<br>CCTCCACCCCCCACGCCGGAAACGCAATAGCCGAAAAACAAAAAACAAAAAAAACAAAAAAAAAACCA<br>AAAAAACAAAACACATTAAAACAGCCTGTGGGTTGATCCCACCCACAGGCCCATTGGGCGCTAGCACT<br>CTGGTATCACGGTACCTTTGTGCGCCTGTTTTATACCCCCTCCCCCAACTGTAACTTAGAAGTAACACAC<br>ACCGATCAACAGTCAGCGTGGCACACCAGCCACGTTTTGATCAAGCACTTCTGTTACCCCGGACTGAGT<br>ATCAATAGACTGCTCACGCGGTTGAAGGAGAAAGCGTTCGTTATCCGGCCAACTACTTCGAAAAACCTA<br>GTAACACCGTGGAAGTTGCAGAGTGTTTCGCTCAGCACTACCCCAGTGTAGATCAGGTCGATGAGTCAC<br>CGCATTCCCCACGGGCGACCGTGGCGGTGGCTGCGTTGGCGGCCTGCCCATGGGGAAACCCATGGGAC<br>GCTCTAATACAGACATGGTGCGAAGAGTCTATTGAGCTAGTTGGTAGTCCTCCGGCCCCTGAATGCGGCT<br>AATCCTAACTGCGGAGCACACACCCTCAAGCCAGAGGGCAGTGTGTCGTAACGGGCAACTCTGCAGCG<br>GAACCGACTACTTTGGGTGTCCGTGTTTCATTTTATTCCTATACTGGCTGCTTATGGTGACAATTGAGAGA<br>TCGTTACCATATAGCTATTGGATTGGCCATCCGGTGACTAATAGAGCTATTATATATCCCTTTGTTGGGTTTA<br>TACCACTTAGCTTGAAAGAGGTTAAAACATTACAATTCATTGTTAAGTTGAATACAGCAAAATGGTGAGC<br>AAGGGCGAGGAGCTGTTCACCGGGGTGGTGCCCATCCTGGTCGAGCTGGACGGCGACGTAAACGGCCA<br>CAAGTTCAGCGTGTCTGGCGAGGGCGAGGGCGATGCCACCTACGGCAAGCTGACCCTGAAGTTCATCT<br>GCACCACCGGCAAGCTGCCCGTGCCCTGGCCCACCCTCGTGACCACCCTGACCTACGGCGTGCAGTGCT<br>TCAGCCGCTACCCCGACCACATGAAGCAGCACGACTTCTTCAAGTCCGCCATGCCCGAAGGCTACGTCC<br>AGGAGCGCACCATCTTCTTCAAGGACGACGGCAACTACAAGACCCGCGCCGAGGTGAAGTTCGAGGGC<br>GACACCCTGGTGAACCGCATCGAGCTGAAGGGCATCGACTTCAAGGAGGACGGCAACATCCTGGGGCA<br>CAAGCTGGAGTACAACTACAACAGCCACAACGTCTATATCATGGCCGACAAGCAGAAGAACGGCATCA<br>AGGCGAACTTCAAGATCCGCCACAACATCGAGGACGGCAGCGTGCAGCTCGCCGACCACTACCAGCAG<br>AACACCCCCATCGGCGACGGCCCCGTGCTGCTGCCCGACAACCACTACCTGAGCACCCAGTCCGCCCTG<br>AGCAAAGACCCCAACGAGAAGCGCGATCACATGGTCCTGCTGGAGTTCGTGACCGCCGCCGGGATCAC<br>TCTCGGCATGGACGAGCTGTACAAGTAAAAAAAACAAAAAACAAAACGGCTATTATGCGTTACCGGCG

	AGACGCTACGGACTTAAATAATTGAGCCTTAAAGAAGAAATTCTTTAAGTGGATGCTCTCAAACTCAGG<br>GAAACCTAAATCTAGTTATAGACAAGGCAATCCTGAGCCAAGCCGAAGTAGTAATTAGTAAGACCAGTG<br>GACAATCGACGGATAACAGCATATCTAGCTGTGCCTTCTAGTTGC
**PIE-CVB3-GFP-mut (unspliced linear precursor)**<br><br>**(The mutation site is indicated in bold and underlined)**	GGGGGAGACCCTCGACCGTCGATTGTCCACTGGTCAACAATAGATGACTTACAACTAATCGGAAGGTGC<br>AGA**T**ACTCGACGGGAGCTACCCTAACGTCAAGACGAGGGTAAAGAGAGAGTCCAATTCTCAAAGCCAA<br>TAGGCAGTAGCGAAAGCTGCAAGAGAATGAAAATCCGTTGACCTTAAACGGTCGTGTGGGTTCAAGTC<br>CCTCCACCCCCACGCCGGAAACGCAATAGCCGAAAAACAAAAAACAAAAAAAAACAAAAAAAAAAACCA<br>AAAAAACAAAACACATTAAAACAGCCTGTGGGTTGATCCCACCCACAGGCCCATTGGGCGCTAGCACT<br>CTGGTATCACGGTACCTTTGTGCGCCTGTTTTATACCCCCTCCCCCAACTGTAACTTAGAAGTAACACAC<br>ACCGATCAACAGTCAGCGTGGCACACCAGCCACGTTTTGATCAAGCACTTCTGTTACCCCGGACTGAGT<br>ATCAATAGACTGCTCACGCGGTTGAAGGAGAAAGCGTTCGTTATCCGGCCAACTACTTCGAAAAACCTA<br>GTAACACCGTGGAAGTTGCAGAGTGTTTCGCTCAGCACTACCCCAGTGTAGATCAGGTCGATGAGTCAC<br>CGCATTCCCCACGGGCGACCGTGGCGGTGGCTGCGTTGGCGGCCTGCCCATGGGGAAACCCATGGGAC<br>GCTCTAATACAGACATGGTGCGAAGAGTCTATTGAGCTAGTTGGTAGTCCTCCGGCCCCTGAATGCGGCT<br>AATCCTAACTGCGGAGCACACACCCTCAAGCCAGAGGGCAGTGTGTCGTAACGGGCAACTCTGCAGCG<br>GAACCGACTACTTTGGGTGTCCGTGTTTCATTTTATTCCTATACTGGCTGCTTATGGTGACAATTGAGAGA<br>TCGTTACCATATAGCTATTGGATTGGCCATCCGGTGACTAATAGAGCTATTATATATCCCTTTGTTGGGTTTA<br>TACCACTTAGCTTGAAAGAGGTTAAAACATTACAATTCATTGTTAAGTTGAATACAGCAAAATGGTGAGC<br>AAGGGCGAGGAGCTGTTCACCGGGGTGGTGCCCATCCTGGTCGAGCTGGACGGCGACGTAAACGGCCA<br>CAAGTTCAGCGTGTCTGGCGAGGGCGAGGGCGATGCCACCTACGGCAAGCTGACCCTGAAGTTCATCT<br>GCACCACCGGCAAGCTGCCCGTGCCCTGGCCCACCCTCGTGACCACCCTGACCTACGGCGTGCAGTGCT<br>TCAGCCGCTACCCCGACCACATGAAGCAGCACGACTTCTTCAAGTCCGCCATGCCCGAAGGCTACGTCC<br>AGGAGCGCACCATCTTCTTCAAGGACGACGGCAACTACAAGACCCGCGCCGAGGTGAAGTTCGAGGGC<br>GACACCCTGGTGAACCGCATCGAGCTGAAGGGCATCGACTTCAAGGAGGACGGCAACATCCTGGGGCA<br>CAAGCTGGAGTACAACTACAACAGCCACAACGTCTATATCATGGCCGACAAGCAGAAGAACGGCATCA<br>AGGCGAACTTCAAGATCCGCCACAACATCGAGGACGGCAGCGTGCAGCTCGCCGACCACTACCAGCAG<br>AACACCCCCATCGGCGACGGCCCCGTGCTGCTGCCCGACAACCACTACCTGAGCACCCAGTCCGCCCTG<br>AGCAAAGACCCCAACGAGAAGCGCGATCACATGGTCCTGCTGGAGTTCGTGACCGCCGCCGGGATCAC<br>TCTCGGCATGGACGAGCTGTACAAGTAAAAAAAACAAAAAACAAAACGGCTATTATGCGTTACCGGCG<br>AGACGCTACGGACTTAAATAATTGAGCCTTAAAGAAGAAATTCTTTAAGTGGATGCTCTCAAACTCAGG<br>GAAACCTAAATCTAGTTATAGACAAGGCAATCCTGAGCCAAGCCGAAGTAGTAATTAGTAAGACCAGTG<br>GACAATCGACGGATAACAGCATATCTAGCTGTGCCTTCTAGTTGC
**STS-CVB3-GFP**	GGTGCGCTCTGCCCACTGACGGGCACCGGAGCGATCGCAGATCCTTCGAATTCTCCTTGAGGCTGAAAA<br>AGTTATCAGGCATGCACCTGGTAGCTAGTCTTTAAACCAATAGATTGCATCGGTTTAAAAGGCAAGACCG<br>TCAAATTGCGGGAAAGGGGTCAACAGCCGTTCAGTACCAAGTCTCAGGGGAAACTTTGAGATGGCCTT<br>GCAAAGGGTATGGTAATAAGCTGACGGACATGGTCCTAACCACGCAGCCAAGTCCTAAGTCAACAGATC<br>TTCTGTTGATATGGATGCAGTTCACAGACTAAATGTCGGTCGGGGAAGATGTATTCTTCTCATAAGATATA<br>GTCGGACCTCTCCTTAATGGGAGCTAGCGGATGAAGTGATGCAACACTGGAGCCGCTGGGAACTAATTT<br>GTATGCGAAAGTATATTGATTAGTTTTGGAGTACTCGTCAAGGACGACGGCAAATACAAGACCCGCGCC<br>GTGGTGAAGTTCGAGGGCGACACCCTGGTGAACCGCATCGAGCTGAAGGGCACCGACTTCAAGGAGG<br>ACGGCAACATCCTGGGGCACAAGCTGGAGTACAACTTTAACAGCCACAACGTCTATATCACCGCCGACA<br>AGCAGAAGAACGGCATCAAGGCGAACTTCACCGTGCGCCACAACGTGGAGGACGGCAGCGTGCAGCT<br>CGCCGACCACTACCAGCAGAACACCCCCATCGGCGACGGCCCCGTGCTGCTGCCCGACAACCACTACC<br>TGAGCACCCAGACCGTGCTGAGCAAAGACCCCAACGAGAAGCGCGATCACATGGTCCTGCATGAGTAT<br>GTGAACGCCGCCGGGATCACTTGATAATAGTTAAAACAGCCTGTGGGTTGATCCCACCCACAGGCCCAT<br>TGGGCGCTAGCACTCTGGTATCACGGTACCTTTGTGCGCCTGTTTTATACCCCCTCCCCCAACTGTAACTT<br>AGAAGTAACACACACCGATCAACAGTCAGCGTGGCACACCAGCCACGTTTTGATCAAGCACTTCTGTTA<br>CCCCGGACTGAGTATCAATAGACTGCTCACGCGGTTGAAGGAGAAAGCGTTCGTTATCCGGCCAACTAC<br>TTCGAAAAACCTAGTAACACCGTGGAAGTTGCAGAGTGTTTCGCTCAGCACTACCCCAGTGTAGATCAG<br>GTCGATGAGTCACCGCATTCCCCACGGGCGACCGTGGCGGTGGCTGCGTTGGCGGCCTGCCCATGGGG<br>AAACCCATGGGACGCTCTAATACAGACATGGTGCGAAGAGTCTATTGAGCTAGTTGGTAGTCCTCCGGC<br>CCCTGAATGCGGCTAATCCTAACTGCGGAGCACACACCCTCAAGCCAGAGGGCAGTGTGTCGTAACGG<br>GCAACTCTGCAGCGGAACCGACTACTTTGGGTGTCCGTGTTTCATTTTATTCCTATACTGGCTGCTTATGG<br>TGACAATTGAGAGATCGTTACCATATAGCTATTGGATTGGCCATCCGGTGACTAATAGAGCTATTATATATC<br>CCTTTGTTGGGTTTATACCACTTAGCTTGAAAGAGGTTAAAACATTACAATTCATTGTTAAGTTGAATACA<br>GCAAAACTAGTGCCACCATGGTGAGCAAGGGCGAGGAGCTGTTCACCGGGGTGGTGCCCATCCTGGTC<br>GAGCTGGACGGCGACGTAAACGGCCACAAGTTCAGCGTGCGCGGCGAGGGCGAGGGCGATGCCACCA<br>TTGGCAAGCTGACCCTGAAGTTCATCTGCACCACCGGCAAGCTGCCCGTGCCCTGGCCCACCCTCGTGA<br>CCACCCTGACCTACGGCGTGCAGTGCTTCAGCCGCTACCCCGACCACATGAAGCAGCACGACTTCTTCA<br>AGTCCGCCATGCCCGAAGGCTACGTCCAGGAGCGCACCATCAGCTTCAGGCGGCCGCGAAGGATCTGC<br>GATCGCTCCGGTGCCCGTCAGTGGGCAGAGCGCAGGATCCAAGCTTAGATAACAG

**STSΔP10-CVB3-GFP**	GGTGCGCTCTGCCCACTGACGGGCACCGGAGCGATCGCAGATCCTTCGAATTCTCCTTGAGGCTGAAAA<br>AGTTATCAGGCATGCACCTGGTAGCTAGTCTTTAAACCAATAGATTGCATCGGTTTAAAAGGCAAGACCG<br>TCAAATTGCGGGAAAGGGGTCAACAGCCGTTCAGTACCAAGTCTCAGGGGAAACTTTGAGATGGCCTT<br>GCAAAGGGTATGGTAATAAGCTGACGGACATGGTCCTAACCACGCAGCCAAGTCCTAAGTCAACAGATC<br>TTCTGTTGATATGGATGCAGTTCACAGACTAAATGTCGGTCGGGGAAGATGTATTCTTCTCATAAGATATA<br>GTCGGACCTCTCCTTAATGGGAGCTAGCGGATGAAGTGATGCAACACTGGAGCCGCTGGGAACTAATTT<br>GTATGCGAAAGTATATTGATTAGTTTTGGAGTACTCGTCAAGGACGACGGCAAATACAAGACCCGCGCC<br>GTGGTGAAGTTCGAGGGCGACACCCTGGTGAACCGCATCGAGCTGAAGGGCACCGACTTCAAGGAGG<br>ACGGCAACATCCTGGGGCACAAGCTGGAGTACAACTTTAACAGCCACAACGTCTATATCACCGCCGACA<br>AGCAGAAGAACGGCATCAAGGCGAACTTCACCGTGCGCCACAACGTGGAGGACGGCAGCGTGCAGCT<br>CGCCGACCACTACCAGCAGAACACCCCCATCGGCGACGGCCCCGTGCTGCTGCCCGACAACCACTACC<br>TGAGCACCCAGACCGTGCTGAGCAAAGACCCCAACGAGAAGCGCGATCACATGGTCCTGCATGAGTAT<br>GTGAACGCCGCCGGGATCACTTGATAATAGTTAAAACAGCCTGTGGGTTGATCCCACCCACAGGCCCAT<br>TGGGCGCTAGCACTCTGGTATCACGGTACCTTTGTGCGCCTGTTTTATACCCCCTCCCCCAACTGTAACTT<br>AGAAGTAACACACACCGATCAACAGTCAGCGTGGCACACCAGCCACGTTTTGATCAAGCACTTCTGTTA<br>CCCCGGACTGAGTATCAATAGACTGCTCACGCGGTTGAAGGAGAAAGCGTTCGTTATCCGGCCAACTAC<br>TTCGAAAAACCTAGTAACACCGTGGAAGTTGCAGAGTGTTTCGCTCAGCACTACCCCAGTGTAGATCAG<br>GTCGATGAGTCACCGCATTCCCCACGGGCGACCGTGGCGGTGGCTGCGTTGGCGGCCTGCCCATGGGG<br>AAACCCATGGGACGCTCTAATACAGACATGGTGCGAAGAGTCTATTGAGCTAGTTGGTAGTCCTCCGGC<br>CCCTGAATGCGGCTAATCCTAACTGCGGAGCACACACCCTCAAGCCAGAGGGCAGTGTGTCGTAACGG<br>GCAACTCTGCAGCGGAACCGACTACTTTGGGTGTCCGTGTTTCATTTTATTCCTATACTGGCTGCTTATGG<br>TGACAATTGAGAGATCGTTACCATATAGCTATTGGATTGGCCATCCGGTGACTAATAGAGCTATTATATATC<br>CCTTTGTTGGGTTTATACCACTTAGCTTGAAAGAGGTTAAAACATTACAATTCATTGTTAAGTTGAATACA<br>GCAAAACTAGTGCCACCATGGTGAGCAAGGGCGAGGAGCTGTTCACCGGGGTGGTGCCCATCCTGGTC<br>GAGCTGGACGGCGACGTAAACGGCCACAAGTTCAGCGTGCGCGGCGAGGGCGAGGGCGATGCCACCA<br>TTGGCAAGCTGACCCTGAAGTTCATCTGCACCACCGGCAAGCTGCCCGTGCCCTGGCCCACCCTCGTGA<br>CCACCCTGACCTACGGCGTGCAGTGCTTCAGCCGCTACCCCGACCACATGAAGCAGCACGACTTCTTCA<br>AGTCCGCCATGCCCGAAGGCTACGTCCAGGAGCGCACCATCAGCT

Table S1. The sequence of transcribed precursors

**DNA probe for RNase H cleavage**	**Probe sequence (5' to 3')**
J16_PIE	CGGATTTTAAGTCCGT
J20_PIE	AACGGATTTTAAGTCCGTAG
J24_PIE	TCAACGGATTTTAAGTCCGTAGCG
J16_STSΔP10	GTCCTTGAAGCTGATG
J20_STSΔP10	TCGTCCTTGAAGCTGATGGT
J24_STSΔP10	CGTCGTCCTTGAAGCTGATGGTGC
J20_T4lig2	GGGTTTCCCCAAACATGGGC
P-GFP	TAGGTCAGGGTGGTCACGAG

Table S2. The sequence of DNA probes for RNase H assay
